# Facile Functionalization of Carbon Electrodes for Efficient Electroenzymatic Hydrogen Production

**DOI:** 10.1021/jacsau.2c00551

**Published:** 2023-01-12

**Authors:** Yongpeng Liu, Sophie Webb, Pavel Moreno-García, Amogh Kulkarni, Plinio Maroni, Peter Broekmann, Ross D. Milton

**Affiliations:** ‡ Department of Inorganic and Analytical Chemistry, University of Geneva, Faculty of Sciences, Quai Ernest-Ansermet 30, Geneva 4 1211, Switzerland; ⊥ National Centre of Competence in Research (NCCR) Catalysis, University of Geneva, Quai Ernest-Ansermet 30, Geneva 4 1211, Switzerland; || Department of Chemistry, Biochemistry and Pharmaceutical Sciences, University of Bern, Freiestrasse 3, Bern 3012, Switzerland; # National Centre of Competence in Research (NCCR) Catalysis, University of Bern, Freiestrasse 3, Bern 3012, Switzerland

**Keywords:** hydrogenase, hydrogen, indium tin oxide, electrode modification, enzymatic electrocatalysis

## Abstract

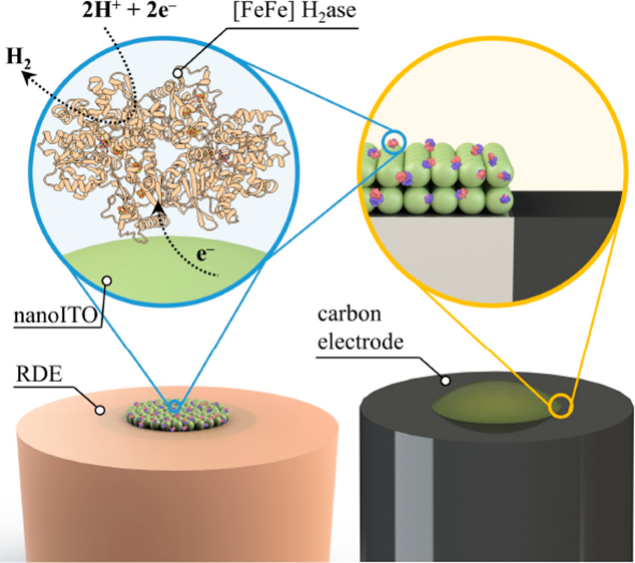

Enzymatic electrocatalysis holds promise for new biotechnological approaches to produce chemical commodities such as molecular hydrogen (H_2_). However, typical inhibitory limitations include low stability and/or low electrocatalytic currents (low product yields). Here we report a facile single-step electrode preparation procedure using indium–tin oxide nanoparticles on carbon electrodes. The subsequent immobilization of a model [FeFe]-hydrogenase from *Clostridium pasteurianum* (“CpI”) on the functionalized carbon electrode permits comparatively large quantities of H_2_ to be produced in a stable manner. Specifically, we observe current densities of >8 mA/cm^2^ at −0.8 V vs the standard hydrogen electrode (SHE) by direct electron transfer (DET) from cyclic voltammetry, with an onset potential for H_2_ production close to its standard potential at pH 7 (approximately −0.4 V vs. SHE). Importantly, hydrogenase-modified electrodes show high stability retaining ∼92% of their electrocatalytic current after 120 h of continuous potentiostatic H_2_ production at −0.6 V vs. SHE; gas chromatography confirmed ∼100% Faradaic efficiency. As the bioelectrode preparation method balances simplicity, performance, and stability, it paves the way for DET on other electroenzymatic reactions as well as semiartificial photosynthesis.

Hydrogen (H_2_) is an energy carrier that has advantages such as high energy density and carbon neutrality. However, present H_2_ production is energy intensive with the majority of the 90 million tons of H_2_ created in 2020^1^ being produced from steam reforming natural gas. Alternatively, electrocatalytic water splitting powered by renewable energy offers a promising approach for sustainable H_2_ production. Despite decades of efforts in developing synthetic electrocatalysts, noble metals are still found to be the most active electrocatalysts for hydrogen evolution reaction (HER).^2,3^ In contrast to synthetic electrocatalysts, however, nature has evolved hydrogenases to specifically catalyze proton reduction and H_2_ oxidation.^4^ Scientists have thus been developing semiartificial approaches to produce H_2_ by enzymatic electrocatalysis (an electrode provides electrons to hydrogenase for proton reduction) to benefit from the unique enzymatic properties such as near-zero overpotential under mild conditions (neutral pH, ambient temperature).^5,6^

Establishing electron transfer between electrodes and oxidoreductase enzymes for electrocatalytic turnover has been a major research focus, where two concepts (mediated electron transfer (MET) and direct electron transfer (DET)) have been developed.^7−9^ The use of redox polymers for hydrogenase immobilization on electrodes is well established and they hold the benchmark current density for H_2_ oxidation (14 mA/cm^2^),^10^ while typically operating with low overpotentials.^11^ In contrast to MET (requiring a diffusive or tethered electron mediator), DET provides a simpler solution to conduct electrons directly between electrodes and enzymes.^12−16^ Wiring oxidoreductase enzymes to electrode surfaces permits heterogeneous substrate turnover and paves the way to study thermodynamic and kinetics properties of enzymes using a variety of electrochemical techniques.^17−19^ Over the past years, numerous efforts have been made to interface hydrogenases with conventional electrodes such as glassy carbon electrodes (GCE),^20^ pyrolytic graphite edge electrodes (PGE),^21,22^ and gold electrodes.^23^ However, poor enzyme loading and nonspecific interactions often significantly limit electrocatalysis, resulting in a current density in the order of μA/cm^2^.

Functionalizing conventional electrodes has therefore long been considered as an effective strategy to improve the performance and stability of hydrogenase electrochemistry. To this end, various methods have employed covalent immobilization,^24^ electrostatic interactions,^25^ and increased surface roughness electrodes^26^ to increase performances. In contrast to the large family of commercially available carbon-based electrodes, metal oxide electrodes have recently emerged as promising candidates for hydrogenase electrochemistry due to their ability to adsorb hydrogenase in a stable and electroactive orientation.^27,28^ Among them, titanium oxide (TiO_2_) and indium tin oxide (ITO) are of particular interest as emerging platforms for enzymatic electrocatalysis, owing to their numerous advantages such as biocompatibility, transparency, earth abundance, chemical/electrochemical stability, and morphological malleability. Compared with TiO_2_, a wide band gap semiconductor with excellent optoelectronic properties, ITO is a type of transparent conducting oxide that has been extensively used in research and industry. The conductivity and accessibility make ITO a good choice as an electrode material.

To the best of our knowledge, there are only 5 reports on the coupling of hydrogenase with ITO electrodes for DET; importantly, the Reisner group has adopted inverse opal ITO electrodes for [NiFeSe]-hydrogenase in 4 reports, where the observed current density ranges from 0.5 to 4.7 mA/cm^2^ at −0.6 V vs. the standard hydrogen electrode (SHE).^29−32^ Recently, Fischer and co-workers electrografted planar ITO electrodes for covalent immobilization of [NiFe]-hydrogenase, where a maximum current density of 0.02 mA/cm^2^ has been reported for H_2_ oxidation at +0.43 V vs. the reversible hydrogen electrode (RHE).^33^ Despite being promising, the complexity of fabricating inverse opal electrodes as well as the low current density on covalently immobilized electrodes still limits the application of ITO to hydrogenase electrocatalysis. In this work, we report a simple method to functionalize conventional carbon electrodes with ITO nanoparticles (“nanoITO”) for efficient, stable, and selective electroenzymatic H_2_ production with [FeFe]-hydrogenase from *Clostridium pasteurianum* (“CpI”). We demonstrate the applicability of this approach using various electrodes such as GCE, PGE, and rotating disk electrodes (RDE), which all yield large and durable current densities. Gas chromatographic (GC) analysis reveals a near-unity Faradaic efficiency (FE) for H_2_ evolution.

Motivated by establishing a simple electrode functionalization process, we adapted and modified earlier reported methods.^34,35^ In brief (the detailed experimental procedure can be found in the Supporting Information), a suspension of ITO nanoparticles (“nanoITO”) was sonicated and drop cast onto electrode surfaces, followed by a mild annealing step at 80 °C in air for 20 min, which can easily be performed in chemistry laboratories. Once cool, 5 μL of hydrogenase (∼25 μg/0.4 nmol per 3 mm GCE) was drop cast onto the nanoITO functionalized electrode and dried at room temperature (electrode optimization on different drop cast volumes can be found in Figure S1 and Table S1) before being evaluated for electrocatalytic H_2_ production (Scheme 1). As shown in Figure 1a, the original mirror-like glassy carbon surface was fully covered by the light green nanoITO film. Morphological characterization including atomic force microscopy (AFM) was performed on nanoITO-GCE in Figure 1b and Figures S2 and S3, where the size of nanoparticle agglomerates were found to be below 50 nm.

**Scheme 1:**
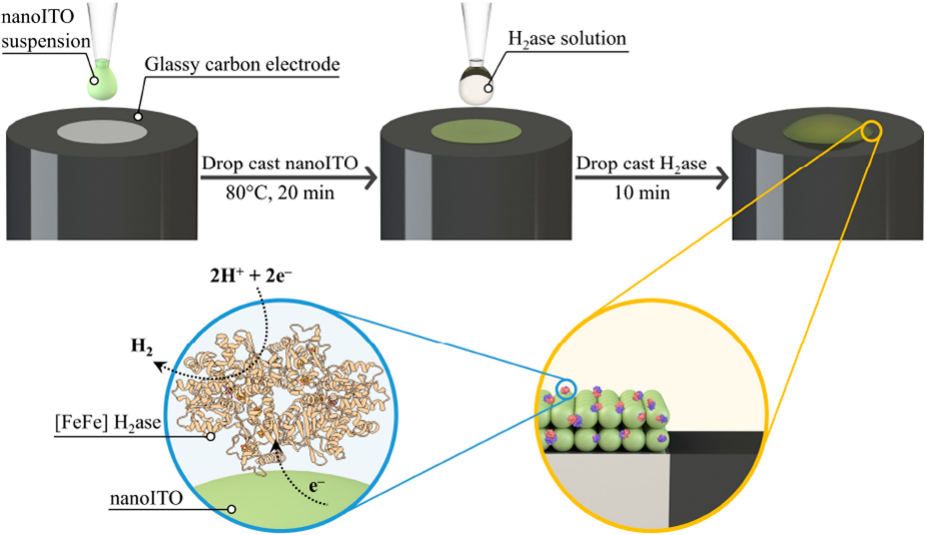
Schematic Illustration of Electrode Functionalization, Hydrogenase Loading, and Electron Transfer/Catalytic Turnover at NanoITO|Hydrogenase|Electrolyte Interfaces

**Figure 1:**
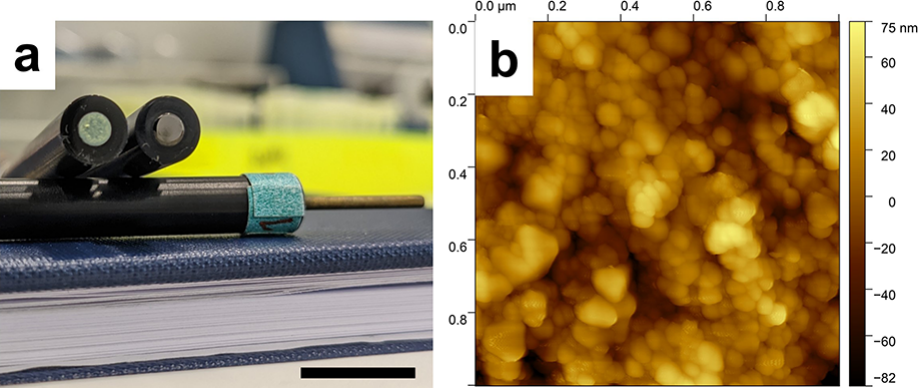
(a) Side-by-side photograph of a nanoITO-modified (left) and unmodified glassy carbon electrodes (scale bar: 10 mm). (b) Atomic force microscopy images of nanoITO-GCE.

We initially utilized cyclic voltammetry (CV) to examine the ability of hydrogenase to undergo DET and produce H_2_ on these nanoITO electrodes. Figure 2a displays a representative CV trace of a nanoITO-hydrogenase GCE in 100 mM MOPS buffer (adjusted to pH 7). The onset potential (defined here as the potential beyond which the catalytic current density exceeds 10 μA/cm^2^) for H_2_ production was observed to be close to the biological standard reduction potential of H_2_ (*E*^0^′ = −0.414 V vs. SHE), with the electrodes therefore exhibiting near zero overpotential for the hydrogen evolution reaction (HER). Surprisingly, we observed a large catalytic current density of 8.7 mA/cm^2^ at −0.8 V vs SHE by CV (7.8 ± 0.7 mA/cm^2^ according to 5 different electrodes in Figure S4), representing comparatively efficient electroenzymatic H_2_ production at this potential (Table S2). The functionalization process was also extended to PGE to verify the compatibility of the method on other conventional electrodes. As shown in Figure 2a, nanoITO-hydrogenase PGEs exhibit current densities of approximately 8.22 mA/cm^2^ at −0.8 V vs. SHE, confirming the reproducibility of this method on other electrodes and the importance of the nanoITO for efficient electrocatalysis (repeat experiments reported in Figure S4). Note that the blank scans without hydrogenase show capacitive behavior with current densities on the order of hundreds of μA/cm^2^, with the significant capacitive current being an indicator of the high surface area of nanoITO modified electrodes. Using double-layer capacitance, we determined the surface area enhancement offered by the use of nanoITO to be 19 on glassy carbon electrodes (Figure S5; note that this is an estimate due to differences in specific capacitances of nanoITO and the underlying GC electrode); accounting for this enhancement yields a corrected electrocatalytic current density that remains >38× the current densities typically obtained on GC electrodes (>6× those typically obtained on PGEs) (Figure S6), reflecting the importance of ITO for efficient [FeFe]-hydrogenase (CpI) immobilization and orientation (improved H_2_ production is not only due to increased electrode surface area). Control experiments on O_2_-deactivated hydrogenase from Figure S7 show >80% current loss at −0.8 V vs. SHE, further confirming that the electrocatalytic current originates from hydrogenase and not the nanoITO electrode, consistent with previous O_2_-deactivation experiments.^36^ The bioelectrode performance was also investigated under H_2_ to understand if nanoITO could influence the reversibility of CpI. As described by Fourmond et al.,^37^ the catalytic reversibility of CpI on nanoITO electrodes is assessed by the overpotential requirement (i.e., the driving force is necessary to achieve a significant catalytic turnover in either direction). This can be realized *via* CV, where *E*_eq_ is the Nernst equilibrium potential of the reactant/product couple (here, 2H^+^ and H_2_, *E*_eq_ = *E*^0^′ = −0.414 V vs. SHE) and the reversibility of the enzyme is assessed by the overpotential (|*E*_eq_ – *E*|) required to achieve a significant catalytic turnover in either direction (where *E* represents the applied potential at the working electrode). Figure S8 compares the electroenzymatic activity of CpI/nanoITO electrodes in pH 7.0 MOPS buffer (100 mM) under 1 atm of Ar or 1 atm of H_2_. Following the introduction of H_2_, an oxidative current is observed. More specifically, no net current flows through the electrode when *E* ≈ −0.404 V vs. SHE and an overpotential of 35 mV in either the oxidative or reductive direction results in electroenzymatic current densities of −0.40 mA/cm^2^ and +0.34 mA/cm^2^ for H^+^ reduction and H_2_ oxidation. A deviation of +0.01 V from *E*^0^′ (when *j* = 0) and observable bidirectional catalysis with a small overpotential in either direction is consistent with CpI acting as a reversible bidirectional catalyst on nanoITO electrodes.

**Figure 2:**
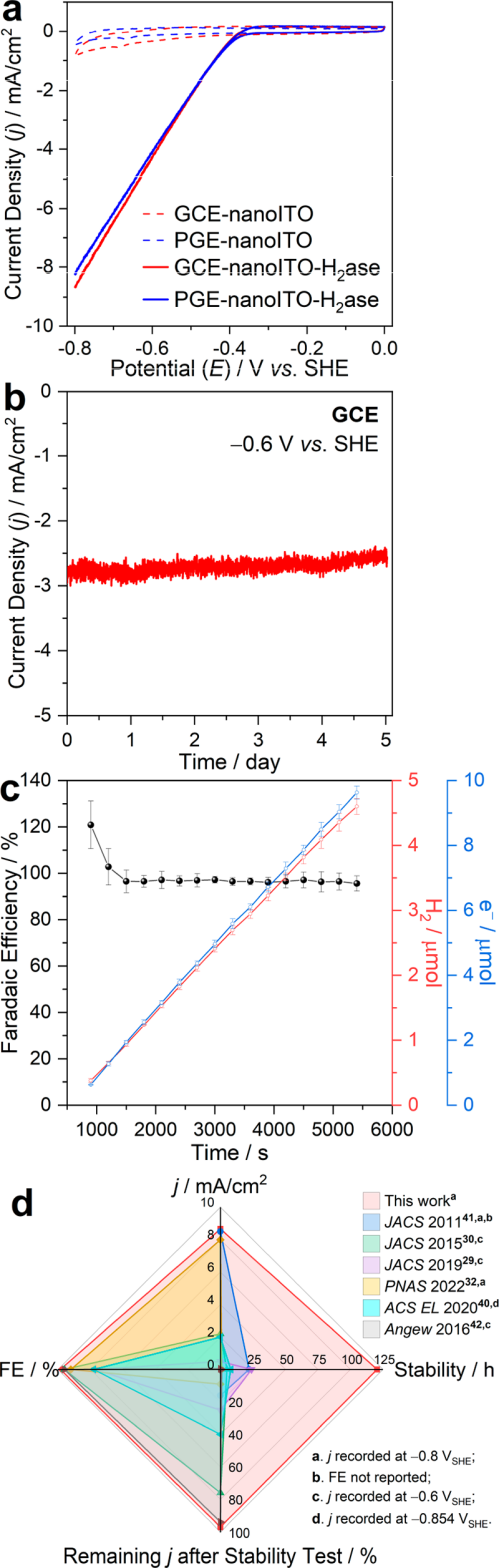
(a) Cyclic voltammetry (third scan, scan rate: 10 mV/s, 5 consecutive scans in Figure S4) of GCE-nanoITO-hydrogenase (red solid line) and PGE-nanoITO-hydrogenase (blue solid line) with corresponding hydrogenase-free electrodes (dashed lines). (b) Amperometric *j*–*t* curve of GCE-nanoITO-hydrogenase at −0.6 V vs SHE over 120 h (5 days) of continuous operation. (c) Online gas chromatography measurement with 1.5 h electrolysis at −0.6 V vs. SHE for Faradaic efficiency (FE) determination (black spheres). Accumulated H_2_ (μmol, red circles) and electrons (blue circles) from online gas chromatography enzymatic electrocatalysis experiments (mean ± standard deviation, *n* = 3). Conditions: Ar-saturated 100 mM MOPS buffer, pH 7, under stirring. (d) Radar plot for comparison of current density (*j*), FE, stability, and remaining *j* after stability test with hydrogenase DET on representative metal oxide electrodes (table of comparison in Table S2).

Subsequently, the stability of electroenzymatic H_2_ production on nanoITO-hydrogenase GCE was evaluated by amperometric *j*–t transients (Figure 2b). At a mild applied potential of −0.6 V vs. SHE, the hydrogenase electrode exhibited a current density of 2.8 mA/cm^2^. After 120 h (5 days) of continuous potentiostatic H_2_ production, 94% of the initial current remained (2.6 mA/cm^2^), representing significantly improved stability of hydrogenase electrodes (and electroenzymatic H_2_ production) so far (Figure 2d and Table S2). The dynamic change in local pH plays a crucial role in the electrocatalytic reaction (with the potential to impact both the substrate concentration and the structure/stability of the enzyme), which can either be resolved by finite element modeling^38^ or be monitored by scanning electrochemical microscopy.^39^ However, (i) these experiments were conducted with rapid stirring, (ii) the pH was found only to drift from 7.04 to 7.22 over this 120 h test, and (iii) this decrease in catalytic current cannot be fully restored in fresh buffer (Figure S9), indicating that the most plausible cause for the decrease in current is simply deactivation of the hydrogenase over time. Additional stability tests for 280 and 1.5 h can be found in Figure S9. The gradual decrease of stability could be a result of fast proton depletion at a high current density. The desorption of hydrogenase from nanoITO electrodes was studied using the Bradford protein assay. As shown in Figure S10, less than 10% of the deposited hydrogenase was detected in the electrolyte after 30 min. This value being larger than the typical decrease in catalytic current over the same period of time suggests that this is the desorption of “excess” hydrogenase that is not undergoing DET. It should be noted that hydrogenases exhibit high selectivity for HER *in vivo*, and (while not particularly expected here) hydrogenase electrochemistry can suffer from film loss, denaturation, and background electrode reactions, which could result in Faradaic efficiencies (FE) below 100%.^29,32,40−42^ To verify the FE of this nanoITO-hydrogenase system, H_2_ production was monitored by an online closed-loop GC system during 1.5 h of enzymatic electrocatalysis at −0.6 V vs. SHE (Figure 2b and c), leading to an average FE of 98.5 ± 3.6% (error = standard deviation, detailed FE calculation provided in the Supporting Information, GC setup and calibration curve in Figures S11 and 12). The superior performance of this electrode architecture can be visualized as a radar plot in Figure 2d (additional references and details can be found in Table S2), keeping in mind the simplicity of electrode fabrication. The FE after 5 days of continuous operation was also found to be ∼103%, during which time the importance of efficient agitation of the electrochemical cell for long-term H_2_ production was identified (Figure S13 and discussion in the Supporting Information).

With the aim of studying reaction kinetics beyond mass transport limitations, we determined whether a kinetic isotope effect (KIE) for electroenzymatic HER was detectable by introducing deuterium oxide (D_2_O) to the electrolyte. We first identified the mass transport region by performing amperometry on RDE-nanoITO-hydrogenase electrodes with various rotational rates ranging from 200 to 3000 rpm with 200 rpm intervals (Figure 3b). During the first 100 s under stationary conditions, local protons are quickly depleted giving rise to a decay in electrocatalytic current likely due to mass transport limitation. The electrocatalytic current magnitude was observed to increase until 2000 rpm; rotation rates of 2500 rpm were therefore chosen for subsequent analysis. The apparent limitations to the electroenzymatic H_2_ current are hypothesized primarily to be due to limited H^+^ transport within the nanoITO/enzyme film. Importantly, other factors that could contribute to this include (i) restricted release of H_2_ from the nanoITO-hydrogenase 3D electrode, or (ii) improved solvent delivery and wetting within the 3D electrode architecture. As shown in Figure 3c, in the nonmass transport limiting region (2500 rpm), the catalytic current in electrolyte containing 50% D_2_O (H:D = 1:1) is lower than that in 100% H_2_O. Likewise, amperometric analysis in Figure 3d clearly indicates 35% electrocatalytic current loss upon the addition of D_2_O (50% final concentration) at 250 s. The observed KIE is in line with our previous work,^36^ and KIE = *i*_H_2_O_/*i*_D_2_O_ ≠ 1, indicating that DET to hydrogenase on these nanoITO electrodes is not rate-limiting for the overall reaction. Conversely, this may also reflect rate-limiting proton-coupled electron transfer (PCET) within this hydrogenase.^43^ Further, differences in mass-transport of H/D within the nanoITO network may also contribute toward this apparent KIE. Detailed analysis on identifying the rate-determining step using KIE is out of scope of this work and has been reported by a series of seminal works elsewhere.^44−47^ Nevertheless, the RDE-nanoITO-hydrogenase reported herein now permits the evaluation of reaction kinetics at a high catalytic turnover rate (current density).

**Figure 3:**
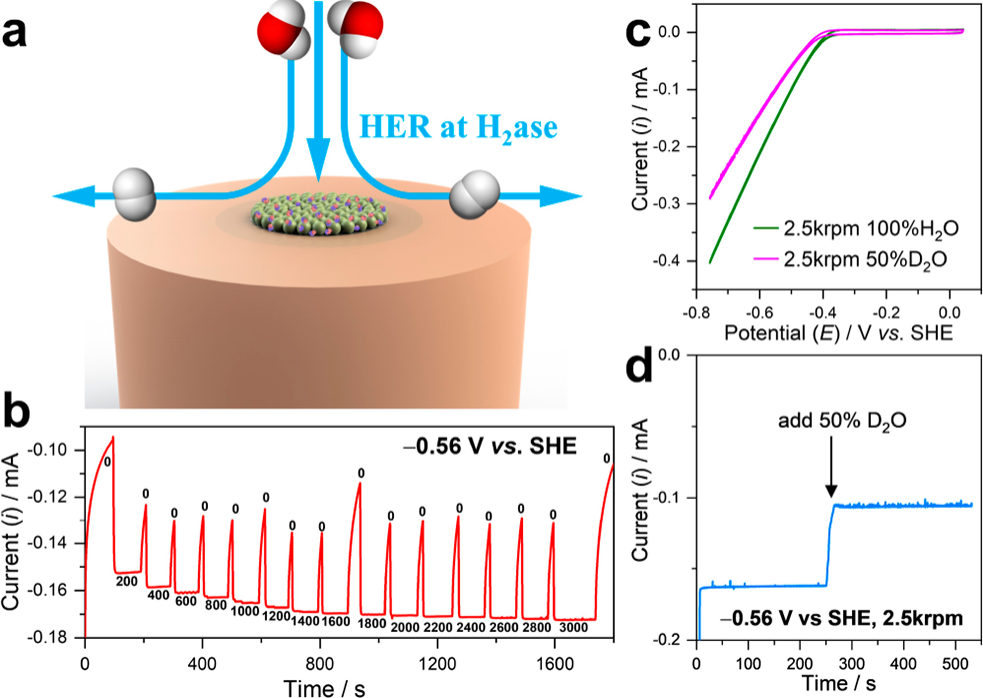
(a) Illustration of RDE-nanoITO-hydrogenase under working conditions. (b) Amperometric *i*–*t* experiment of RDE-nanoITO-hydrogenase with different rotation rate (rpm) at −0.56 V vs. SHE. (c) Cyclic voltammetry (second scan) of RDE-nanoITO-hydrogenase at 2500 rpm, before and after adding 50% D_2_O. (d) Amperometric *i*–*t* at −0.56 V vs. SHE, 2500 rpm, before and after adding 50% D_2_O.

Finally, the nanoITO-hydrogenase system was evaluated using a novel inverted RDE setup coupled to online gas chromatography (iRDE-GC) (Figures S14 and 15) to simultaneously evaluate the performance, stability, and FE during galvanostatic H_2_ production^48^ under well-defined convective conditions with applied current densities of −1.53, −2.55, and −3.56 mA/cm^2^ (iRDE-GC performed at 500 rpm). Video S1 illustrating the setup of this system is provided as Supporting Information. As shown in Figure 4a, the nanoITO-hydrogenase iRDE-GC system undergoes stable galvanostatic electrolysis for 1 h, further confirming the applicability of the nanoITO functionalization method. Note that the potential gradually becomes more negative over time to sustain chronopotentiometry at −3.56 mA/cm^2^, which may indicate reductive inactivation of [FeFe]-hydrogenases.^49^ However, we cannot rule-out O_2_-based deactivation introduced by performing this experiment on the bench with Ar-purging. Key advantages of the iRDE-GC setup include (i) improved mass transfer due to laminar flow of the substrates across the electrode surface, and (ii) a gastight configuration where the H_2_ yield can be continuously monitored; the real-time FE of galvanostatic electrolysis is shown in Figure 4a (solid spheres). In the first 15 min, the FE gradually increases toward ca. 100%, clearly indicating dissolution of H_2_ in electrolyte and the equilibration of H_2_ in the headspace of the cell. Upon H_2_ saturation, a near-unity FE can be observed from 20 to 60 min. Similarly, potentiostatic electrolysis was performed in this iRDE-GC system at −0.53, −0.59, and −0.64 V vs. SHE. As shown in Figure 4b, stable current densities can be observed for more than 1 h with close to 100% FE following H_2_ saturation. The application of nanoITO in the iRDE-GC setup represents a novel platform for enzyme electrochemistry with high performance, durability, and no side reactions.

**Figure 4:**
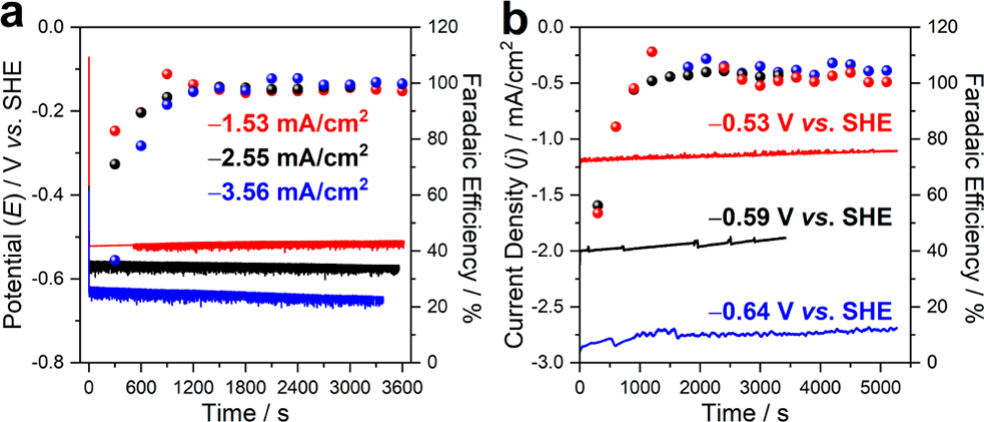
(a) Galvanostatic electrolysis of iRDE-GC nanoITO-hydrogenase system at −1.53, −2.55, and −3.56 mA/cm^2^ with corresponding FE. (b) Potentiostatic electrolysis of iRDE-GC nanoITO-hydrogenase at −0.53, −0.59, and −0.64 V vs. SHE with corresponding FE (experiments performed at 500 rpm).

In conclusion, we report a simple one-step method to functionalize conventional electrodes with nanoITO for enzymatic electrocatalysis. Taking [FeFe]-hydrogenase as a model enzyme, CV and amperometric analysis reveal large electrocatalytic current densities (8.66 mA/cm^2^ at −0.8 V vs. SHE) and high stabilities (maintaining 94% of the initial current after 120 h), and online GC measurements confirm near-ideal FEs of 98.5 ± 3.6% for electroenzymatic H_2_ production, owing to the high porosity and electroactive interaction between metal oxide and hydrogenase. In addition, nanoITO functionalized RDE and iRDE allow the study of KIE, mass transport, and FE at a high catalytic turnover rate (in terms of gross electroenzymatic H_2_ produced at an electrode surface) and a stable condition with no observable side reactions. While this method establishes a new benchmark for electroenzymatic H_2_ production, we anticipate that it has the potential to be applied to other electroenzymatic systems such as formate dehydrogenase for carbon dioxide reduction and nitrogenase for ammonia production, enabling large quantities of enzymes to be immobilized. It could also be rationally extended to photoelectrodes, with the proper adjustment of film thickness for transparency, to perform artificial photosynthesis for solar to chemical conversion.

## Data Availability

All raw data are available on the Zenodo repository (10.5281/zenodo.6641837).

## Supporting Information Available

Full description of the experimental methods, [FeFe]-hydrogenase preparation, electrode optimization, representative AFM images, repeats of hydrogenase electrochemistry, electrochemical active surface area (ECSA), O_2_-deactivation control experiment, additional stability tests, hydrogenase desorption, GC measurements and FE determination, iRDE setup, SDS-PAGE (PDF)
Video S1, setup of the nanoITO-hydrogenase system (MP4)

## References

[ref1] Bermudez J.; Hasegawa T.; Bennett S. Hydrogen – Analysis and Key Findings. A Report by the International Energy Agency. 2021. https://www.iea.org/reports/hydrogen (accessed 2022-04-20).

[ref2] Lin L.; Ge Y.; Zhang H.; Wang M.; Xiao D.; Ma D. Heterogeneous Catalysis in Water. JACS Au 2021, 1 (11), 1834–1848. 10.1021/jacsau.1c00319. 34841403 PMC8611672

[ref3] Zhu J.; Hu L.; Zhao P.; Lee L. Y. S.; Wong K.-Y. Recent Advances in Electrocatalytic Hydrogen Evolution Using Nanoparticles. Chem. Rev. 2020, 120 (2), 851–918. 10.1021/acs.chemrev.9b00248. 31657904

[ref4] Lubitz W.; Ogata H.; Rüdiger O.; Reijerse E. Hydrogenases. Chem. Rev. 2014, 114 (8), 4081–4148. 10.1021/cr4005814. 24655035

[ref5] Armstrong F. A.; Belsey N. A.; Cracknell J. A.; Goldet G.; Parkin A.; Reisner E.; Vincent K. A.; Wait A. F. Dynamic Electrochemical Investigations of Hydrogen Oxidation and Production by Enzymes and Implications for Future Technology. Chem. Soc. Rev. 2009, 38 (1), 36–51. 10.1039/B801144N. 19088963

[ref6] Cracknell J. A.; Vincent K. A.; Armstrong F. A. Enzymes as Working or Inspirational Electrocatalysts for Fuel Cells and Electrolysis. Chem. Rev. 2008, 108 (7), 2439–2461. 10.1021/cr0680639. 18620369

[ref7] Léger C.; Bertrand P. Direct Electrochemistry of Redox Enzymes as a Tool for Mechanistic Studies. Chem. Rev. 2008, 108 (7), 2379–2438. 10.1021/cr0680742. 18620368

[ref8] Xiao X.; Xia H.; Wu R.; Bai L.; Yan L.; Magner E.; Cosnier S.; Lojou E.; Zhu Z.; Liu A. Tackling the Challenges of Enzymatic (Bio)Fuel Cells. Chem. Rev. 2019, 119 (16), 9509–9558. 10.1021/acs.chemrev.9b00115. 31243999

[ref9] Ruff A.; Conzuelo F.; Schuhmann W. Bioelectrocatalysis as the Basis for the Design of Enzyme-Based Biofuel Cells and Semi-Artificial Biophotoelectrodes. Nat. Catal. 2020, 3 (3), 214–224. 10.1038/s41929-019-0381-9.

[ref10] Szczesny J.; Birrell J. A.; Conzuelo F.; Lubitz W.; Ruff A.; Schuhmann W. Redox-Polymer-Based High-Current-Density Gas-Diffusion H_2_-Oxidation Bioanode Using [FeFe] Hydrogenase from Desulfovibrio Desulfuricans in a Membrane-Free Biofuel. Cell. Angew. Chem. Int. Ed. 2020, 59 (38), 16506–16510. 10.1002/anie.202006824. PMC7540381 32432842

[ref11] Hardt S.; Stapf S.; Filmon D. T.; Birrell J. A.; Rüdiger O.; Fourmond V.; Léger C.; Plumeré N. Reversible H_2_ Oxidation and Evolution by Hydrogenase Embedded in a Redox Polymer Film. Nat. Catal. 2021, 4 (3), 251–258. 10.1038/s41929-021-00586-1. 33842839 PMC7610533

[ref12] Milton R. D.; Minteer S. D. Direct Enzymatic Bioelectrocatalysis: Differentiating between Myth and Reality. J. R. Soc. Interface 2017, 14 (131), 20170253 10.1098/rsif.2017.0253. 28637918 PMC5493807

[ref13] Hambourger M.; Gervaldo M.; Svedruzic D.; King P. W.; Gust D.; Ghirardi M.; Moore A. L.; Moore T. A. [FeFe]-Hydrogenase-Catalyzed H_2_ Production in a Photoelectrochemical Biofuel Cell. J. Am. Chem. Soc. 2008, 130 (6), 2015–2022. 10.1021/ja077691k. 18205358

[ref14] Lalaoui N.; de Poulpiquet A.; Haddad R.; Le Goff A.; Holzinger M.; Gounel S.; Mermoux M.; Infossi P.; Mano N.; Lojou E.; Cosnier S. A Membraneless Air-Breathing Hydrogen Biofuel Cell Based on Direct Wiring of Thermostable Enzymes on Carbon Nanotube Electrodes. Chem. Commun. 2015, 51 (35), 7447–7450. 10.1039/C5CC02166A. 25845356

[ref15] Karyakin A. A.; Morozov S. V.; Voronin O. G.; Zorin N. A.; Karyakina E. E.; Fateyev V. N.; Cosnier S. The Limiting Performance Characteristics in Bioelectrocatalysis of Hydrogenase Enzymes. Angew. Chem., Int. Ed. 2007, 46 (38), 7244–7246. 10.1002/anie.200701096. 17668427

[ref16] Morozov S. V.; Vignais P. M.; Cournac L.; Zorin N. A.; Karyakina E. E.; Karyakin A. A.; Cosnier S. Bioelectrocatalytic Hydrogen Production by Hydrogenase Electrodes. Int. J. Hydrog. Energy 2002, 27 (11), 1501–1505. 10.1016/S0360-3199(02)00091-5.

[ref17] Armstrong F. A.; Evans R. M.; Hexter S. V.; Murphy B. J.; Roessler M. M.; Wulff P. Guiding Principles of Hydrogenase Catalysis Instigated and Clarified by Protein Film Electrochemistry. Acc. Chem. Res. 2016, 49 (5), 884–892. 10.1021/acs.accounts.6b00027. 27104487

[ref18] Vincent K. A.; Parkin A.; Armstrong F. A. Investigating and Exploiting the Electrocatalytic Properties of Hydrogenases. Chem. Rev. 2007, 107 (10), 4366–4413. 10.1021/cr050191u. 17845060

[ref19] Léger C.; Elliott S. J.; Hoke K. R.; Jeuken L. J. C.; Jones A. K.; Armstrong F. A. Enzyme Electrokinetics: Using Protein Film Voltammetry To Investigate Redox Enzymes and Their Mechanisms. Biochemistry 2003, 42 (29), 8653–8662. 10.1021/bi034789c. 12873124

[ref20] Bianco P.; Haiadjian J. Electrocatalysis at Hydrogenase or Cytochrorme C3-Modified Glassy Carbon Electrodes. Electroanalysis 1991, 3 (9), 973–977. 10.1002/elan.1140030915.

[ref21] Fourmond V.; Greco C.; Sybirna K.; Baffert C.; Wang P.-H.; Ezanno P.; Montefiori M.; Bruschi M.; Meynial-Salles I.; Soucaille P.; Blumberger J.; Bottin H.; De Gioia L.; Léger C. The Oxidative Inactivation of FeFe Hydrogenase Reveals the Flexibility of the H-Cluster. Nat. Chem. 2014, 6 (4), 336–342. 10.1038/nchem.1892. 24651202

[ref22] Pandey K.; Islam S. T. A.; Happe T.; Armstrong F. A. Frequency and Potential Dependence of Reversible Electrocatalytic Hydrogen Interconversion by [FeFe]-Hydrogenases. Proc. Natl. Acad. Sci. U. S. A. 2017, 114 (15), 3843–3848. 10.1073/pnas.1619961114. 28348243 PMC5393252

[ref23] Krassen H.; Stripp S.; von Abendroth G.; Ataka K.; Happe T.; Heberle J. Immobilization of the [FeFe]-Hydrogenase CrHydA1 on a Gold Electrode: Design of a Catalytic Surface for the Production of Molecular Hydrogen. J. Biotechnol. 2009, 142 (1), 3–9. 10.1016/j.jbiotec.2009.01.018. 19480942

[ref24] Rüdiger O.; Abad J. M.; Hatchikian E. C.; Fernandez V. M.; De Lacey A. L. Oriented Immobilization of Desulfovibrio Gigas Hydrogenase onto Carbon Electrodes by Covalent Bonds for Nonmediated Oxidation of H_2_. J. Am. Chem. Soc. 2005, 127 (46), 16008–16009. 10.1021/ja0554312. 16287271

[ref25] Badiani V. M.; Cobb S. J.; Wagner A.; Oliveira A. R.; Zacarias S.; Pereira I. A. C.; Reisner E. Elucidating Film Loss and the Role of Hydrogen Bonding of Adsorbed Redox Enzymes by Electrochemical Quartz Crystal Microbalance Analysis. ACS Catal. 2022, 12 (3), 1886–1897. 10.1021/acscatal.1c04317. 35573129 PMC9097293

[ref26] Wang Y.; Kang Z.; Zhang L.; Zhu Z. Elucidating the Interactions between a [NiFe]-Hydrogenase and Carbon Electrodes for Enhanced Bioelectrocatalysis. ACS Catal. 2022, 12 (2), 1415–1427. 10.1021/acscatal.1c05306.

[ref27] Reisner E.; Powell D. J.; Cavazza C.; Fontecilla-Camps J. C.; Armstrong F. A. Visible Light-Driven H_2_ Production by Hydrogenases Attached to Dye-Sensitized TiO_2_ Nanoparticles. J. Am. Chem. Soc. 2009, 131 (51), 18457–18466. 10.1021/ja907923r. 19928857

[ref28] Reisner E.; Fontecilla-Camps J. C.; Armstrong F. A. Catalytic Electrochemistry of a [NiFeSe]-Hydrogenase on TiO_2_ and Demonstration of Its Suitability for Visible-Light Driven H_2_ Production. Chem. Commun. 2009, 5, 550–552. 10.1039/B817371K. 19283287

[ref29] Sokol K. P.; Robinson W. E.; Oliveira A. R.; Zacarias S.; Lee C.-Y.; Madden C.; Bassegoda A.; Hirst J.; Pereira I. A. C.; Reisner E. Reversible and Selective Interconversion of Hydrogen and Carbon Dioxide into Formate by a Semiartificial Formate Hydrogenlyase Mimic. J. Am. Chem. Soc. 2019, 141 (44), 17498–17502. 10.1021/jacs.9b09575. 31638793 PMC6838786

[ref30] Mersch D.; Lee C.-Y.; Zhang J. Z.; Brinkert K.; Fontecilla-Camps J. C.; Rutherford A. W.; Reisner E. Wiring of Photosystem II to Hydrogenase for Photoelectrochemical Water Splitting. J. Am. Chem. Soc. 2015, 137 (26), 8541–8549. 10.1021/jacs.5b03737. 26046591

[ref31] Lee C.-Y.; Reuillard B.; Sokol K. P.; Laftsoglou T.; Lockwood C. W. J.; Rowe S. F.; Hwang E. T.; Fontecilla-Camps J. C.; Jeuken L. J. C.; Butt J. N.; Reisner E. A Decahaem Cytochrome as an Electron Conduit in Protein–Enzyme Redox Processes. Chem. Commun. 2016, 52 (46), 7390–7393. 10.1039/C6CC02721K. 27193068

[ref32] Edwardes Moore E.; Cobb S. J.; Coito A. M.; Oliveira A. R.; Pereira I. A. C.; Reisner E. Understanding the Local Chemical Environment of Bioelectrocatalysis. Proc. Natl. Acad. Sci. U. S. A. 2022, 119 (4), e2114097119 10.1073/pnas.2114097119. 35058361 PMC8795565

[ref33] Harris T. G. A. A.; Heidary N.; Frielingsdorf S.; Rauwerdink S.; Tahraoui A.; Lenz O.; Zebger I.; Fischer A. Electrografted Interfaces on Metal Oxide Electrodes for Enzyme Immobilization and Bioelectrocatalysis. ChemElectroChem. 2021, 8 (7), 1329–1336. 10.1002/celc.202100020.

[ref34] Hoertz P. G.; Chen Z.; Kent C. A.; Meyer T. J. Application of High Surface Area Tin-Doped Indium Oxide Nanoparticle Films as Transparent Conducting Electrodes. Inorg. Chem. 2010, 49 (18), 8179–8181. 10.1021/ic100719r. 20712331

[ref35] Kornienko N.; Zhang J. Z.; Sokol K. P.; Lamaison S.; Fantuzzi A.; van Grondelle R.; Rutherford A. W.; Reisner E. Oxygenic Photoreactivity in Photosystem II Studied by Rotating Ring Disk Electrochemistry. J. Am. Chem. Soc. 2018, 140 (51), 17923–17931. 10.1021/jacs.8b08784. 30188698 PMC6311681

[ref36] Khushvakov J.; Nussbaum R.; Cadoux C.; Duan J.; Stripp S. T.; Milton R. D. Following Electroenzymatic Hydrogen Production by Rotating Ring–Disk. Electrochemistry and Mass Spectrometry. Angew. Chem. Int. Ed. 2021, 60 (18), 10001–10006. 10.1002/anie.202100863. 33630389

[ref37] Fourmond V.; Plumeré N.; Léger C. Reversible Catalysis. Nat. Rev. Chem. 2021, 5 (5), 348–360. 10.1038/s41570-021-00268-3. 37117844

[ref38] Cobb S. J.; Badiani V. M.; Dharani A. M.; Wagner A.; Zacarias S.; Oliveira A. R.; Pereira I. A. C.; Reisner E. Fast CO_2_ Hydration Kinetics Impair Heterogeneous but Improve Enzymatic CO_2_ Reduction Catalysis. Nat. Chem. 2022, 14 (4), 417–424. 10.1038/s41557-021-00880-2. 35228690 PMC7612589

[ref39] Monteiro M. C. O.; Mirabal A.; Jacobse L.; Doblhoff-Dier K.; Barton S. C.; Koper M. T. M. Time-Resolved Local PH Measurements during CO_2_ Reduction Using Scanning Electrochemical Microscopy: Buffering and Tip Effects. JACS Au 2021, 1 (11), 1915–1924. 10.1021/jacsau.1c00289. 34849509 PMC8611793

[ref40] Edwardes Moore E.; Andrei V.; Zacarias S.; Pereira I. A. C.; Reisner E. Integration of a Hydrogenase in a Lead Halide Perovskite Photoelectrode for Tandem Solar Water Splitting. ACS Energy Lett. 2020, 5 (1), 232–237. 10.1021/acsenergylett.9b02437. 32010793 PMC6986817

[ref41] Svedružić D.; Blackburn J. L.; Tenent R. C.; Rocha J.-D. R.; Vinzant T. B.; Heben M. J.; King P. W. High-Performance Hydrogen Production and Oxidation Electrodes with Hydrogenase Supported on Metallic Single-Wall CarbonNanotube Networks. J. Am. Chem. Soc. 2011, 133 (12), 4299–4306. 10.1021/ja104785e. 21384925

[ref42] Lee C.-Y.; Park H. S.; Fontecilla-Camps J. C.; Reisner E. Photoelectrochemical H_2_ Evolution with a Hydrogenase Immobilized on a TiO_2_-Protected Silicon Electrode. Angew. Chem., Int. Ed. 2016, 55 (20), 5971–5974. 10.1002/anie.201511822. PMC4981910 27061334

[ref43] Lampret O.; Duan J.; Hofmann E.; Winkler M.; Armstrong F. A.; Happe T. The Roles of Long-Range Proton-Coupled Electron Transfer in the Directionality and Efficiency of [FeFe]-Hydrogenases. Proc. Natl. Acad. Sci. U. S. A. 2020, 117 (34), 20520–20529. 10.1073/pnas.2007090117. 32796105 PMC7456106

[ref44] Ratzloff M. W.; Wilker M. B.; Mulder D. W.; Lubner C. E.; Hamby H.; Brown K. A.; Dukovic G.; King P. W. Activation Thermodynamics and H/D Kinetic Isotope Effect of the Hox to HredH+ Transition in [FeFe] Hydrogenase. J. Am. Chem. Soc. 2017, 139 (37), 12879–12882. 10.1021/jacs.7b04216. 28851216

[ref45] Abou Hamdan A.; Dementin S.; Liebgott P.-P.; Gutierrez-Sanz O.; Richaud P.; De Lacey A. L.; Rousset M.; Bertrand P.; Cournac L.; Léger C. Understanding and Tuning the Catalytic Bias of Hydrogenase. J. Am. Chem. Soc. 2012, 134 (20), 8368–8371. 10.1021/ja301802r. 22540997

[ref46] Leroux F.; Dementin S.; Burlat B.; Cournac L.; Volbeda A.; Champ S.; Martin L.; Guigliarelli B.; Bertrand P.; Fontecilla-Camps J.; Rousset M.; Léger C. Experimental Approaches to Kinetics of Gas Diffusion in Hydrogenase. Proc. Natl. Acad. Sci. U. S. A. 2008, 105 (32), 11188–11193. 10.1073/pnas.0803689105. 18685111 PMC2516266

[ref47] Yang H.; Gandhi H.; Cornish A. J.; Moran J. J.; Kreuzer H. W.; Ostrom N. E.; Hegg E. L. Isotopic Fractionation Associated with [NiFe]- and [FeFe]-Hydrogenases. Rapid Commun. Mass Spectrom. 2016, 30 (2), 285–292. 10.1002/rcm.7432. 27071219

[ref48] Moreno-Garcia P.; Kovacs N.; Grozovski V.; Galvez-Vazquez M. d. J.; Vesztergom S.; Broekmann P. Toward CO_2_ Electroreduction under Controlled Mass Flow Conditions: A Combined Inverted RDE and Gas Chromatography Approach. Anal. Chem. 2020, 92 (6), 4301–4308. 10.1021/acs.analchem.9b04999. 32081004 PMC7307836

[ref49] Hajj V.; Baffert C.; Sybirna K.; Meynial-Salles I.; Soucaille P.; Bottin H.; Fourmond V.; Léger C. FeFe Hydrogenase Reductive Inactivation and Implication for Catalysis. Energy Environ. Sci. 2014, 7 (2), 715–719. 10.1039/C3EE42075B.

