# Supplementary material for: Facile Functionalization of Carbon Electrodes for Efficient Electroenzymatic Hydrogen Production
Source: JACS Au. 2023 Jan 12;3(1):124–30. doi: 10.1021/jacsau.2c00551 (PMC9875370; doi:10.1021/jacsau.2c00551)
Supplement: Supplementary file 2 — au2c00551_si_002.pdf [file au2c00551_si_002.pdf]

Supporting Information

Facile functionalization of carbon electrodes for efficient electroenzymatic hydrogen production

Yongpeng Liu,[1,†,‡*] Sophie Webb,[1,2, ‡] Pavel Moreno-García,[3] Amogh Kulkarni,[1] Plinio Maroni,[1] Peter Broekmann,[3,4] and Ross D. Milton[1,2,*]

[1]Department of Inorganic and Analytical Chemistry, University of Geneva, Faculty of Sciences, Quai Ernest-Ansermet 30, 1211 Geneva 4, Switzerland

[2]National Centre of Competence in Research (NCCR) Catalysis, University of Geneva, Quai Ernest-Ansermet 30, 1211 Geneva 4, Switzerland

[3]Department of Chemistry, Biochemistry and Pharmaceutical Sciences, University of Bern, Freiestrasse 3, 3012 Bern, Switzerland

[4]National Centre of Competence in Research (NCCR) Catalysis, University of Bern, Freiestrasse 3, 3012 Bern, Switzerland

Corresponding Author

E-mail: ross.milton@unige.ch
E-mail: yl862@cam.ac.uk

Present Addresses

†Yusuf Hamied Department of Chemistry, University of Cambridge, Lensfield Road, Cambridge, CB2 1EW, United Kingdom.

Author Contributions

‡These authors contributed equally.

Contents

S1 Materials and methods S4

S1.1 [FeFe]-hydrogenase preparation S4

S1.2 Preparation of nanoITO functionalized electrode S5

S1.3 Electrochemical measurements S6

S2 Electrode optimization S7

S3 Atomic force microscopy (AFM) S8

S4 Repeats of hydrogenase electrochemistry S10

S5 Electrochemical active surface area (ECSA) S11

S6 Hydrogenase electrochemistry without nanoITO S12

S7 O_2-deactivation control experiments S14

S8 H_2 oxidation experiments S15

S9 List of state of the art hydrogenase electrochemistry S16

S10 Additional stability tests S17

S11 Bradford protein assay for hydrogenase leaching S18

S12 Gas chromatography (GC) measurements and Faradaic efficiency (FE) determination S19

S13 Long-term Faradaic efficiency of the CpI-nanoITO bioelectrode S21

S14 Inverted rotating disk electrode (iRDE) setup S23

S15 SDS-PAGE S25

References S26

List of Figures

S1 Electrode optimization . . . S7

S2 Representative AFM image . . . S8

S3 AFM image of scratched nanoITO electrode . . . S9

S4 Repeats of hydrogenase electrochemistry . . . S10

S5 Electrochemical active surface area (ECSA) measurements . . . S11

S6 Hydrogenase electrochemistry without nanoITO . . . S13

S7 O_2-deactivation control experiments . . . S14

S8 H_2 oxidation experiments . . . S15

S9 Additional stability tests . . . S17

S10 Bradford protein assay for hydrogenase leaching . . . S18

S11 Sketch of GC setup . . . S19

S12 Linear response of the GC setup . . . S20

S13 Long-term Faradaic efficiency of the CpI-nanoITO bioelectrode . . . S22

S14 Inverted RDE (iRDE) and electrochemical cell assembly . . . S23

S15 Schematic of iRDE setup . . . S24

S16 SDS page . . . S25

List of Tables

S1 Optimized drop cast volume for conventional electrodes. . . . S5

S2 List of state of the art hydrogenase electrochemistry. . . . S16

S1 Materials and methods

S1.1 [FeFe]-hydrogenase preparation

[FeFe]-hydrogenase from *Clostridium pasteurianum* ("CpI") was recombinantly produced in *Escherichia coli* (*E. coli*) with a C-terminal Strep-tag, as previously reported; [FeFe]-hydrogenase was matured *in vivo* using a second plasmid containing the HydEFGX genes from *Shewanella oneidensis*.[S1,S2] Following anaerobic overnight expression at 18 °C, cell lysis was performed by sonication within an anoxic chamber (COY laboratories, Michigan). The lysate was clarified by centrifugation (30'000 × g, 30 min) and the supernatant was passed over a StrepTactin-XT column (Cytiva, 5 mL). The lysis buffer contained 2 mM dithionite to maintain reducing conditions during lysis and clarification (the supernatant therefore contained dithionite at 2 mM final concentration). The FPLC purification buffers did not contain dithionite (degassed in the anoxic chamber for > 2 days), and the dithionite within the supernatant was therefore washed upon binding of CpI to the solid-phase. All subsequent steps were performed in the absence of dithionite to yield dithionite-free CpI. [FeFe]-hydrogenase elution was performed with 50 mM biotin, which was subsequently removed with a desalting column (HiPrep 26/10 Desalting). The purified enzyme was concentrated to approximately 5 mg/mL and flash frozen in liquid N_2. The specific activity of [FeFe]-hydrogenase was 135 ± 24 µmol H_2 $min^{-1}mg^{-1}$, as determined by online gas chromatography (GC). The specific activity was determined within an Ar atmosphere anoxic glovebox (Jacomex, France) at 30 °C in MOPS buffer (0.1 M, pH 7) using 1 mM methyl viologen as the electron mediator and 100 mM dithionite as the overall electron donor.

S1.2 Preparation of nanoITO functionalized electrode

The preparation of a stock nanoITO colloidal suspension was modified from the method developed by Meyer and co-workers.[S3] A 20% by weight indium tin oxide nanoparticle (nanoITO, In_2O_3:SnO_2 9:1 wt%, 17–28 nm APS, 99.5%, Thermo Scientific) suspension was prepared in a solution of 5 M acetic acid in absolute ethanol and sonicated for 20 min to ensure homogeneity. The volume of nanoITO suspension drop coated on the electrode was optimized for a given electrode surface area, as shown in Table S1. Note that a good nanoITO film should uniformly cover the whole electrode active area and be pinhole-free (Figure 1a). After the evaporation of solvent (around 1 min), nanoITO coated working electrodes were annealed at 80 °C in air for 20 min to achieve improved contact. Note that in Meyer's procedures,[S3] the nanoITO electrodes were annealed at 500 °C for 1 hour and then at 300 °C under 3% H_2/N_2 for 1 hour, which is modified to 80 °C in air for 20 min to better adapt carbon electrodes.

Table S1: Optimized drop cast volume for conventional electrodes.

Working electrode	Diameter	Volume
Glassy carbon (CHI104, CH Instruments)	3 mm	1.5 µL
Pyrolytic graphite (XJ057 PGE, ALS)	3 mm	1.5 µL
Glassy carbon (disk electrode, Pine Research)	5 mm	3.0 µL
Pyrolytic graphite (disk electrode, Pine Research)	3 mm	1.5 µL

S1.3 Electrochemical measurements

All electrochemical experiments were performed under an Ar atmosphere ($O_2 < 0.1$ ppm, Jacomex, France), using a Metrohm-Autolab biopotentiostat (PGSTAT302N MBA). The 3-electrode configuration in a standard 15 mL cell (Pine Research, RRPG223) consists of a working electrode, a saturated calomel reference electrode (CHI150), and a coiled Pt wire counter electrode. The applied potentials versus saturated calomel electrode (SCE) were converted to the standard hydrogen electrode (SHE) by the following correction: $E_{\mathrm{SHE}} = E_{\mathrm{SCE}} + 0.242$ V.[S4] All working electrodes were thoroughly polished with 0.3 µm diameter α-Al_2O_3 powder slurry on a wool polishing pad (Struers, DP-Mol) prior to nanoITO fictionalization. Unless stated elsewhere, all reported cyclic voltammetry (CV) traces were the third scan using a 10 mV/s scan rate and 1 mV step potential.

0.1 M MOPS buffer (Roth, Germany) was employed for all electrochemical experiments, prepared and pH adjusted with HCl/NaOH aerobically until pH = 7, then stirred under an Ar atmosphere for at least 48 h. For kinetic isotope experiments, a 0.1 M MOPS in D_2O (Eurisotop, Product code: 7789-20-0), was prepared, and pH adjusted with sodium deuteuroxide (NaOD, Sigma) until a pD = 7.41 (pD = 'pH' + 0.41). The electrolyte pH was determined by a pH meter (Metrohm).

Rotating disk electrode (RDE) experiments were performed on an electrode rotator (Pine Research, WaveVortex 10) in an electrochemical cell that containing a Luggin capillary and a fritted counter electrode compartment (Adams & Chittenden Scientific Glass, product code: 957219, USA). The counter electrode (coiled platinum wire) was placed behind either a ceramic or porous glass frit in all RDE and bulk electrolytic experiments.

S2 Electrode optimization

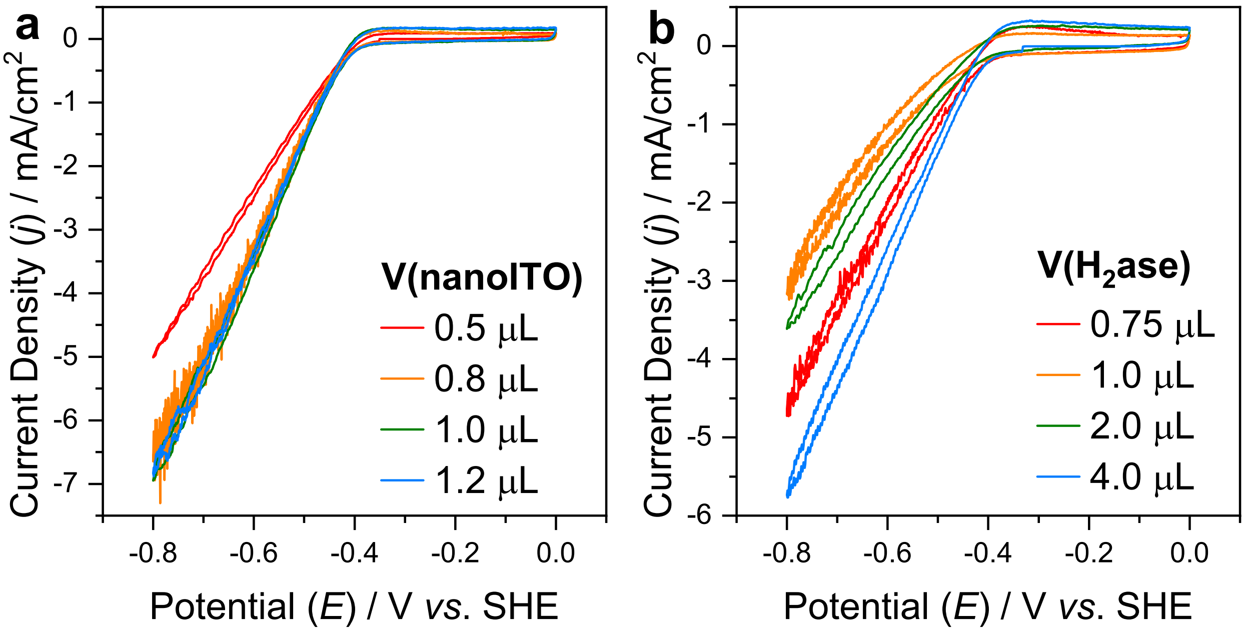

Figure S1: Electrode optimization on (a) nanoITO volume and (b) hydrogenase volume. Cyclic voltammetry of GCE-nanoITO-hydrogenase electrodes (third scan, scan rate: 10 mV/s) with (a) different nanoITO volume with 5 μL hydrogenase and (b) different hydrogenase volume with 1.5 μL nanoITO. Note that 3 mm GCE electrode cannot hold more than 1.5 μL nanoITO.

S3 Atomic force microscopy (AFM)

Images of the nanoITO electrode were acquired in amplitude modulated mode (AM-AFM) with an Infinity MFP3D AFM (Asylum Research, Oxford Instruments). AC240TS cantilevers (Olympus, Japan) were excited close to their resonance frequencies (70 kHz) and with free oscillation amplitudes (FOA) of approximately 160 nm. The set point and the scan rate were around 50% of the FOA and 0.2 Hz respectively. A typical image of a freshly prepared electrode is shown in Figure S2a. Section profiles along the black and red lines are shown in Figure S2b.

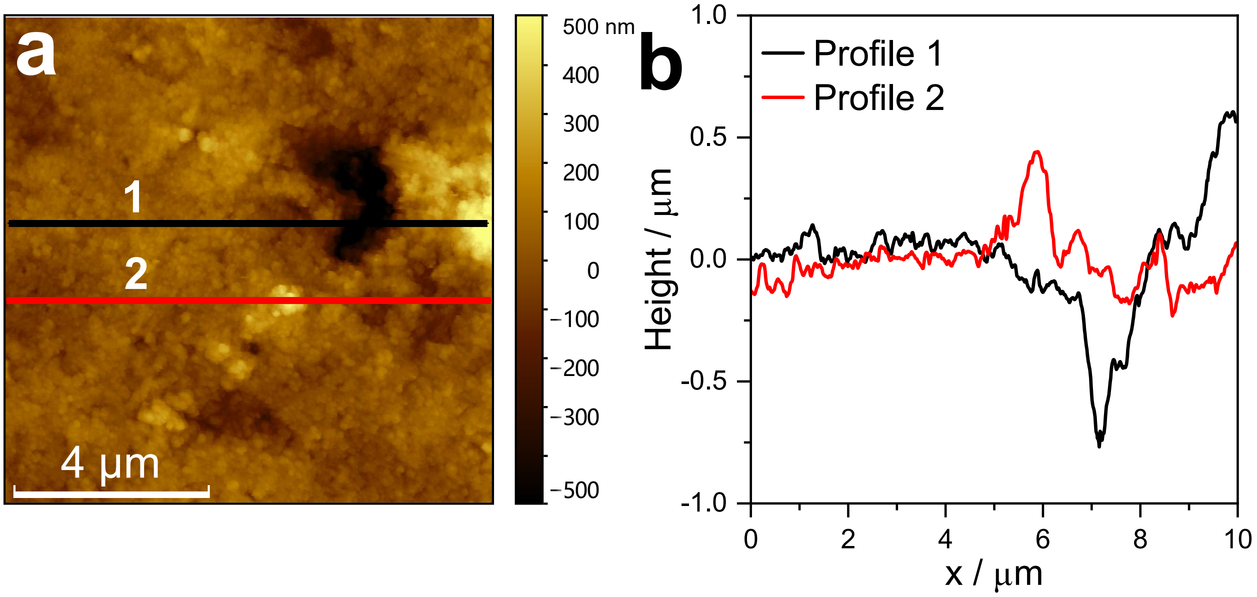

Figure S2: (a) Representative AFM image of a freshly prepared nanoITO electrode. (b) Section profiles along lines 1 (black) and 2 (red).

To determine the thickness of the nanoparticle layer, a freshly prepared nanoITO electrode was scratched with a knife edge to produce a small groove on the surface. Subsequently, an AFM topography is recorded in vicinity of the boundary of the scratch. In Figure S3a the topographic image of such area and in Figure S3b its section profile along the black line are represented. Comparing the heights at the boundary one can extract a layer thickness of around 9 µm.

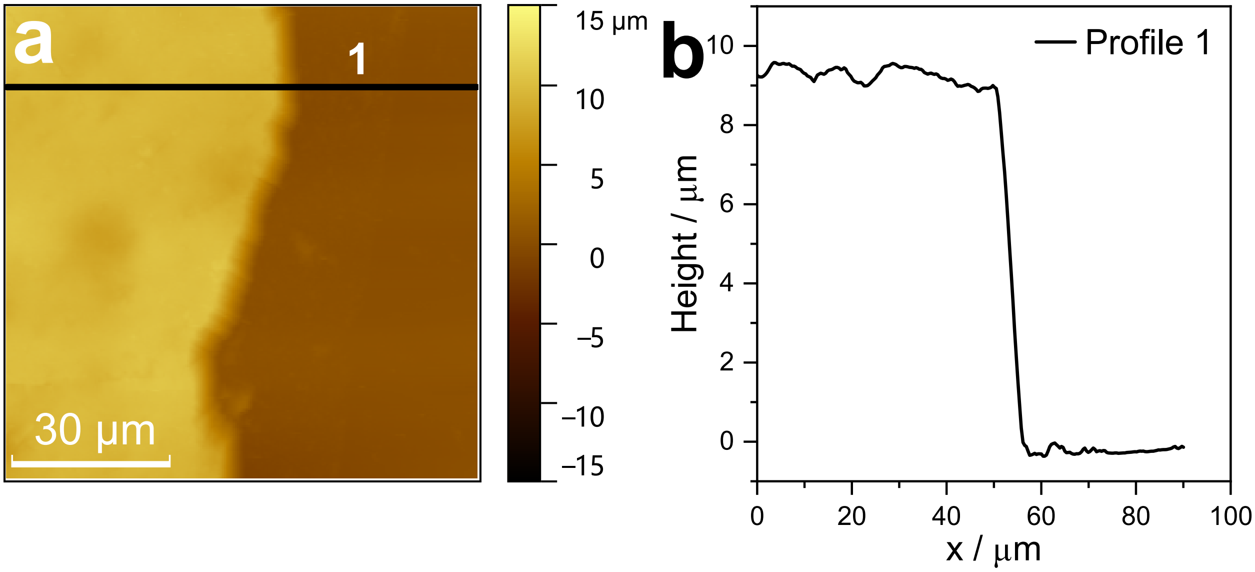

Figure S3: (a) AFM image of scratched nanoITO electrode. (b) Section profile along the black line in (a).

S4 Repeats of hydrogenase electrochemistry

To validate the reproducibility of our reported hydrogenase electrochemistry. We performed CV on 5 different GCE-nanoITO-hydrogenase electrodes in Figure S4a and 2 different PGE-nanoITO-hydrogenase electrodes in Figure S4b. Figure S4c,d show 5 consecutive CV scans on 2 GCE electrodes where all CV traces are reasonably overlapped, confirming the electrochemical properties of the electrodes did not change.

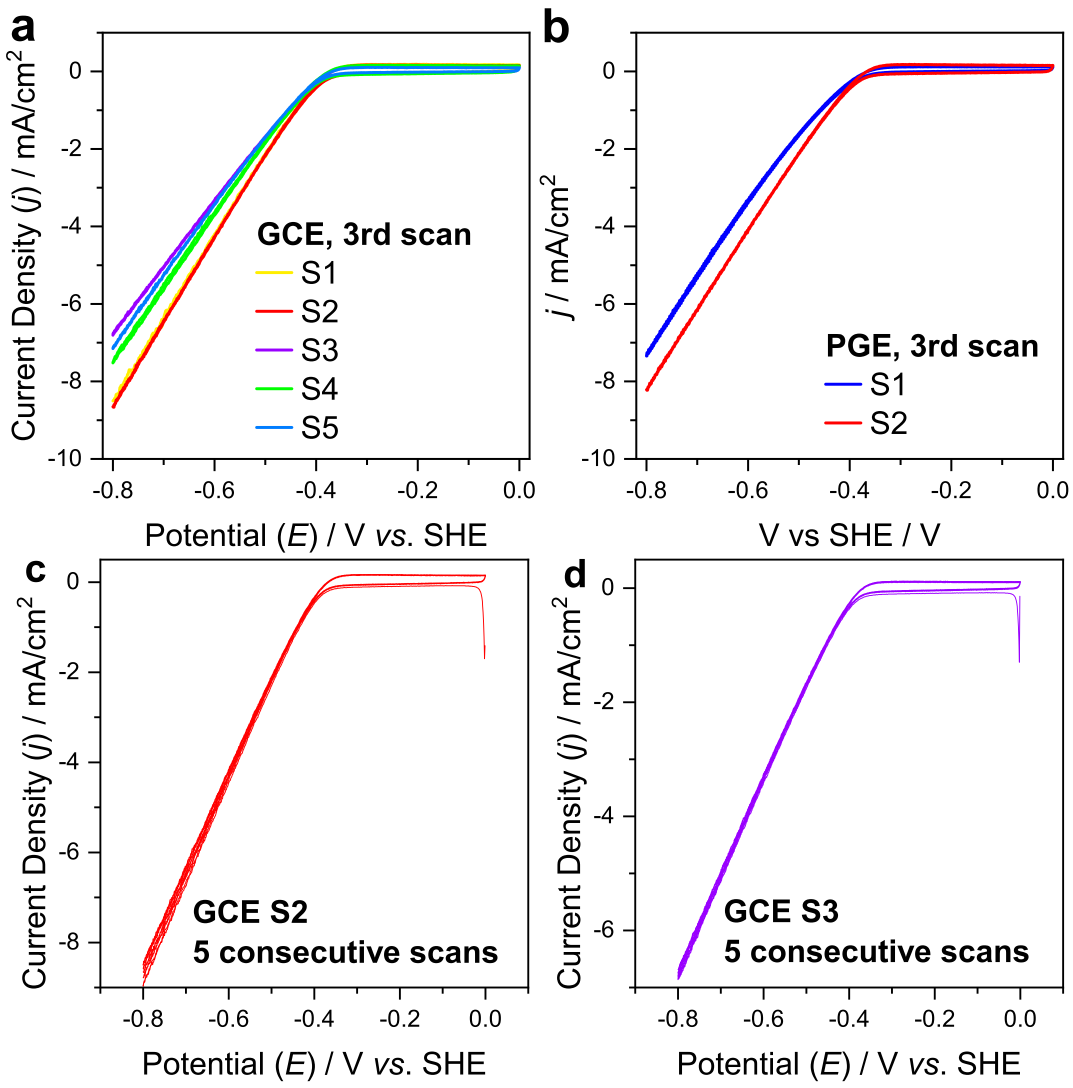

Figure S4: Repeats of (a) GCE-nanoITO-hydrogenase on 5 different electrodes and (b) PGE-nanoITO-hydrogenase on 2 different electrodes where the third CV scan is plotted. (c)-(d) 5 consecutive CV scans on 2 GCE electrodes.

S5 Electrochemical active surface area (ECSA)

Figure S5a-b show the 5th CV scans at non-Faradaic region where only capacitive current is presenting on blank GCE (Figure S5a) and nanoITO (Figure S5b). When plot capacitive current density difference (Δj, taking value at 0 V *vs.* SHE) as a function of scan rate in Figure S5c, a linear relationship is observed. Linear regression reveals a slope of 365 μF/cm^2 for blank GCE and a slope of 6770 μF/cm^2 for nanoITO. The surface roughness factor is estimated by the ratio between the two slopes where a value of 19 is found.

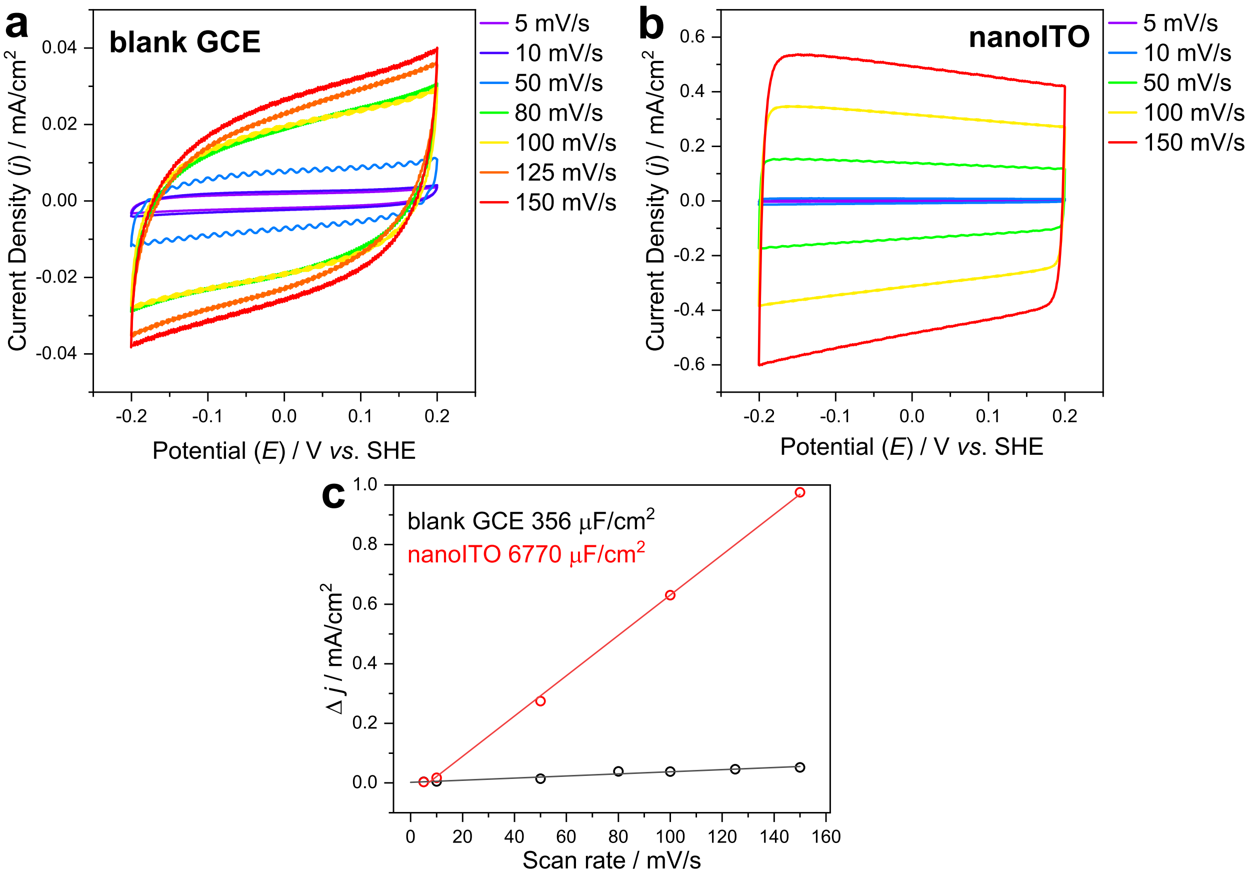

Figure S5: Electrochemical active surface area (ECSA) measurements on (a) blank GCE and (b) nanoITO at different scan rates (5th scan). (c) Capacitive current density difference (Δj) as a function of scan rate.

S6 Hydrogenase electrochemistry without nanoITO

To validate the catalytic current density enhancement is not only as result of surface roughness, hydrogenase electrochemistry has been performed on bare GCE (Figure S6a) and bare PGE (Figure S6c). As a comparison, corresponding nanoITO modified electrodes have been plotted in Figure S6b,d. At −0.8 V *vs.* SHE, current density for each electrode is: 0.0118 mA/cm^2 for GCE-H_2ase, 0.063 mA/cm^2 for PGE-H_2ase, 8.66 mA/cm^2 for GCE-nanoITO-H_2ase, and 8.22 mA/cm^2 for PGE-nanoITO-H_2ase. Considering a surface roughness of 19 from ECSA (Figure S5), corrected current densities for GCE-nanoITO-H_2ase and PGE-nanoITO-H_2ase are 0.456 mA/cm^2 and 0.433 mA/cm^2, respectively. The ratio between corrected current density on nanoITO electrode and current density on bare electrode are 38.6 for GCE and 6.9 for PGE. The fact that the ratio is greater than 1 indicates current density enhancement is not solely due to increased surface area. As a electrode material, nanoITO itself can adsorb hydrogenase in a stable and electroactive orientation.[S5,S6]

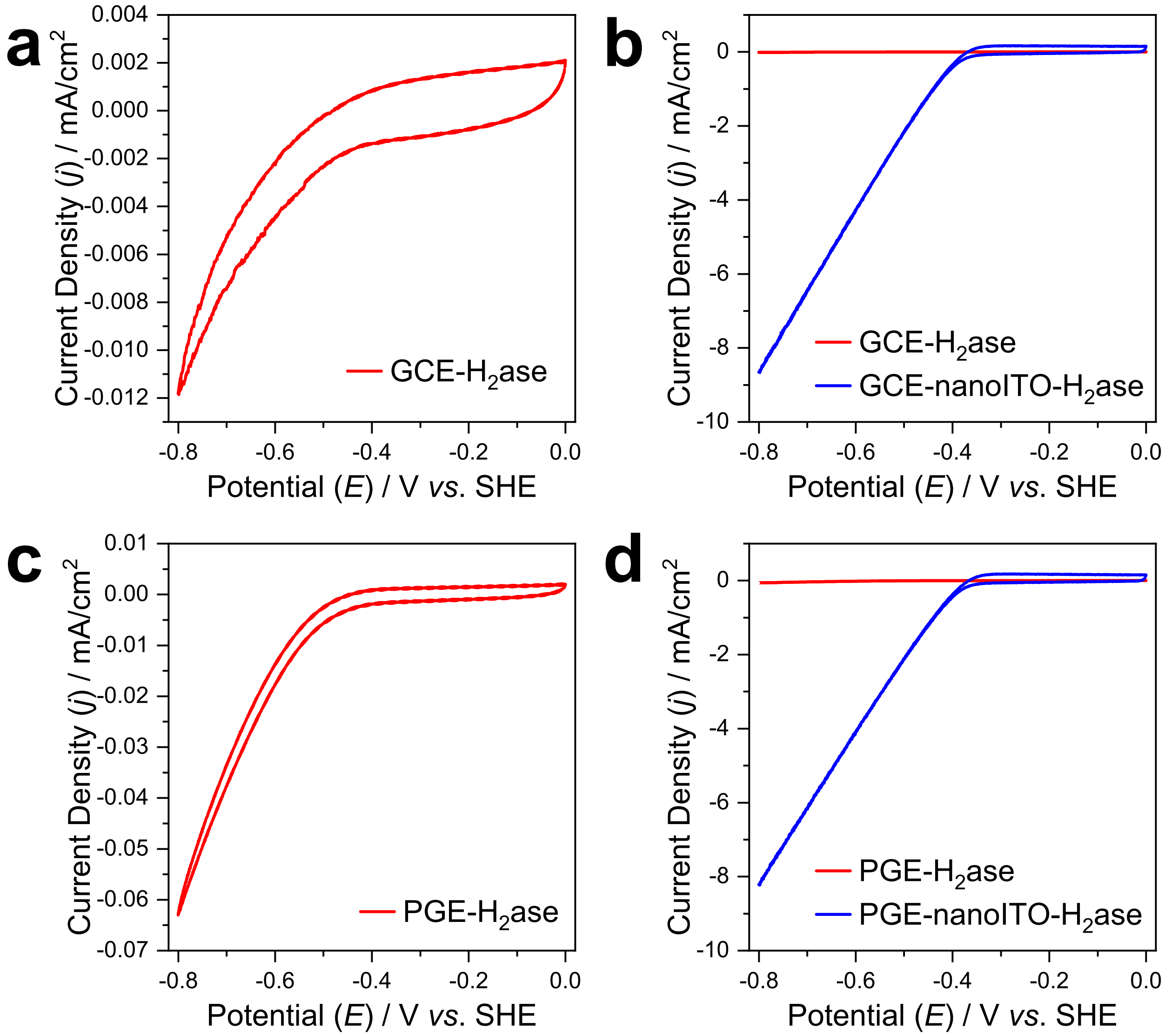

Figure S6: Cyclic voltammetry (third scan, scan rate: 10 mV/s) of (a) GCE-hydrogenase and (c) PGE-hydrogenase with (b), (d) corresponding nanoITO modified electrodes.

S7 O_2-deactivation control experiments

To validate the catalytic current is originated from [FeFe]-hydrogenase instead of nanoITO. A O_2-deactivation control experiment was performed by leaving the GCE-nanoITO-hydrogenase electrode under ambient condition for 20 min. The high oxygen level (around 21%) and the drying process were mean to deactivate the [FeFe]-hydrogenase. As we observed in Figure S7, deactivated bioelectrode shows a significantly lower current density than that of functional bioelectrode, around 17% at −0.8 V *vs.* SHE.

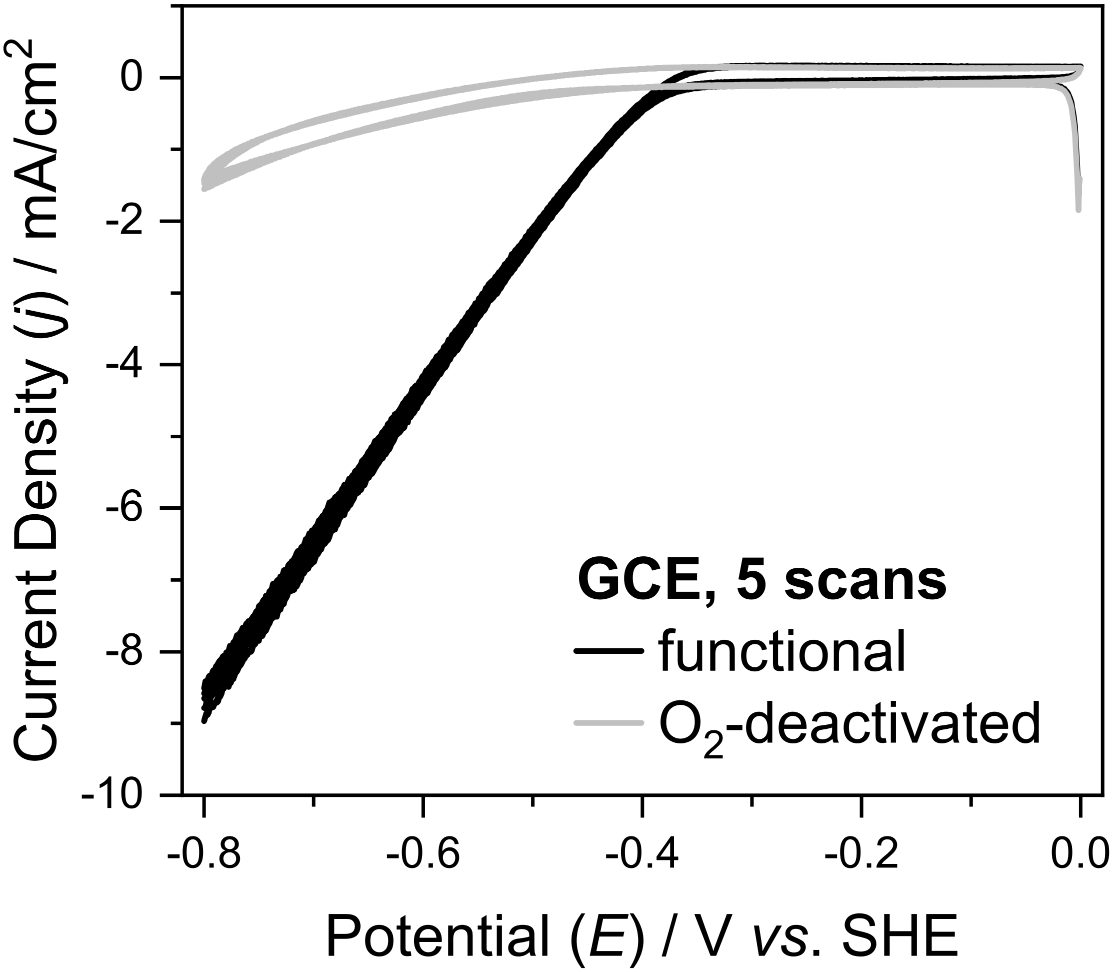

Figure S7: 5 consecutive CV scans of functional and O_2-deactivated GCE-nanoITO-hydrogenase electrode.

S8 H_2 oxidation experiments

A 3 mm GCE nanoITO-hydrogenase electrode was prepared as described in Section S1.2 and dropcast with 5 µL CpI. A gastight electrochemical cell was assembled under Ar, as described in Section S12. H_2 gas was introduced through a needle inserted into the cell septum and gently bubbled into the buffer for 3 min, then vented to ambient pressure. A CV was recorded (conditions in Section S1.3) before and after H_2 addition.

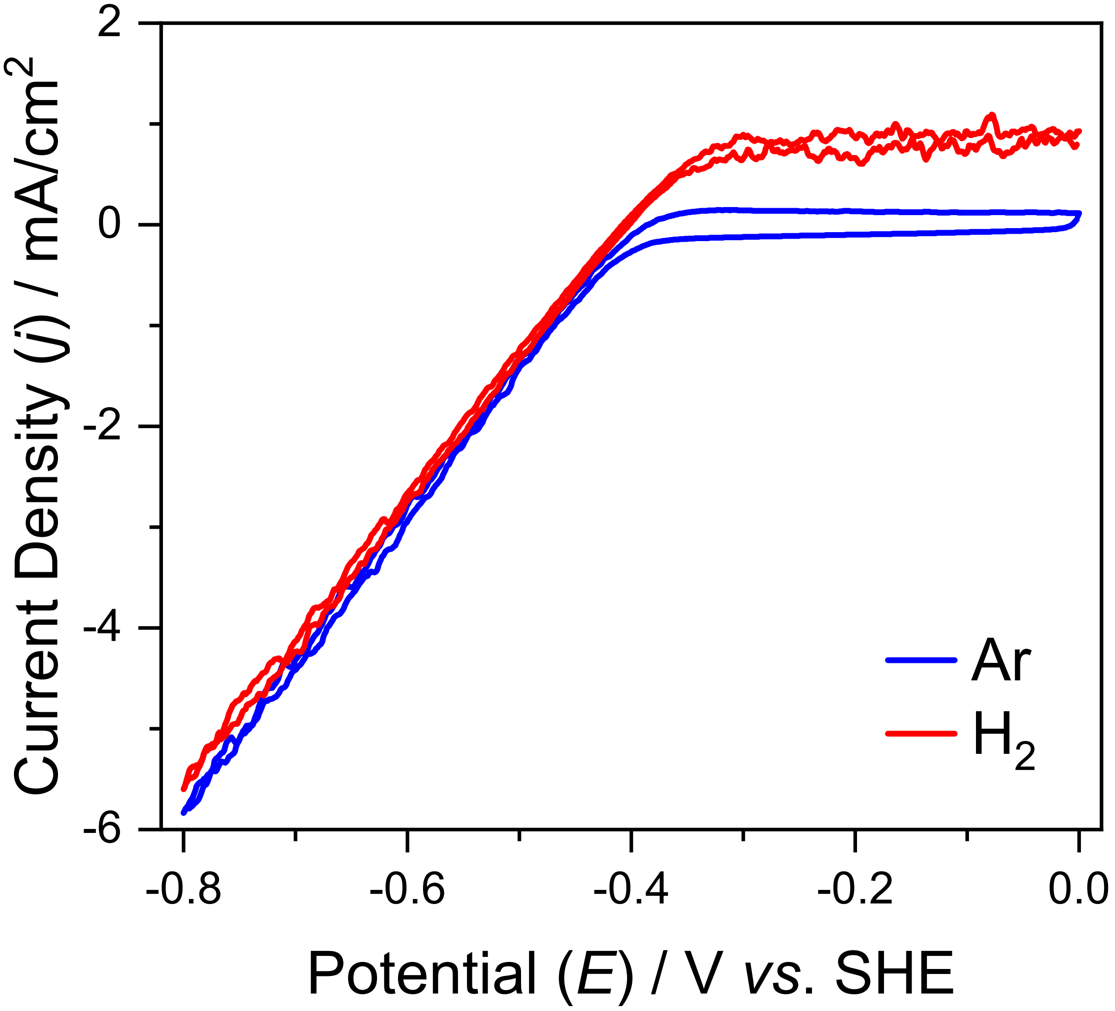

Figure S8: CV scans of GCE-nanoITO-hydrogenase electrode under Ar and H_2.

S9 List of state of the art hydrogenase electrochemistry

Table S2: List of state of the art hydrogenase electrochemistry.

Type	Electrode configuration	Electrolyte	Stability					FE (%)	Reference
			Current density (mA/cm^2) @ potential (V vs SHE)	time (h)	potential (V vs SHE)	initial current (mA/cm^2)	remaining current (mA/cm^2)		
[FeFe]	**GCE|nanoITO**	**100 mM MOPS pH 7**	**8.66 @ –0.8**	**280.5**	**–0.6**	**3.15**	**1.41 (45%)**	**98.5±3.6**	**this work**
				**120.5**	**–0.6**	**2.8**	**2.63 (94%)**		
				**1.5**	**–0.6**	**4.45**	**4.31 (97%)**		
	C-cloth|SWCNT	100 mM TES pH 7	12 @ –1	22	–0.6	3.8	0.6 (16%)	NA	*J. Am. Chem. Soc.* **2011**, *133*, 4299–4306
			8.5 @ –0.8	1.5	–0.6	3.8	2.3 (61%)		
	FTO|meso-TiO_2	50 mM Aces pH 6.8	1.9 @ –0.8	2	–0.741	1.6	0.8 (50%)	98±1	*Bioelectrochemistry*, **2015**, *106*, 258
				1.5	–0.741	1.6	0.96 (60%)		
	FTO|meso-TiO_2	60 mM phosphate pH 6	0.91 @ –0.79	0.5	–0.741	0.45	0.09 (20%)	NA	*Chem. Commun.*, **2011**, *47*, 10566–10568
[NiFe]	ITO|diazonium	10 mM phosphate pH 5.5	0.02 @ +0.1 (HOR)	1.5	+0.1	0.022	0.018 (82%)	NA	*ChemElectroChem* **2021**, *8*, 1329–133
[NiFeSe]	FTO|IO-ITO	40 mM MES pH 6.5	2.2 @ –0.6	5	–0.6	1.8	1.36 (76%)	96±3	*J. Am. Chem. Soc.* **2015**, *137*, 8541−8549
				1.5	–0.6	1.8	1.6 (89%)		
	FTO|IO-ITO	100 mM $NaHCO_3$ pH 6.5	0.5 @ –0.6	24	–0.6	0.6	0.15 (25%)	77	*J. Am. Chem. Soc.* **2019**, *141*, 17498−17502
				1.5	–0.6	0.6	0.25 (42%)		
	FTO|IO-ITO	mixed buffer pH 7.0	0.7 @ –0.7	NA	NA	NA	NA	NA	*Chem. Commun.*, **2016**, *52*, 7390
	Ti|IO-TiO_2	240 mM MES pH 4.24	8 @ –0.8	1	–0.8	8	2.7 (34%)	92	*PNAS* **2022**, *119*, 4 e2114097119
			2.2 @ –0.6	1	–0.6	2.2	0.2 (9%)		
	FTO|mesoITO		4.7 @ –0.6	1	–0.6	4.7	2.1 (45%)		
	Ti|IO-TiO_2	50 mM MES pH 6	2 @ –0.854	8	–0.854	2.5	1 (40%)	78	*ACS Energy Lett.* **2020**, *5*, 1, 232–237
				1.5	–0.854	2.5	2 (80%)		
	FTO|TiO_2	50 mM MES pH 6	0.04 @ –0.6	1	–0.35	0.0065	0.0061 (94%)	96±6	*Angew. Chem.* **2016**, *55*, 5971 –5974

S10 Additional stability tests

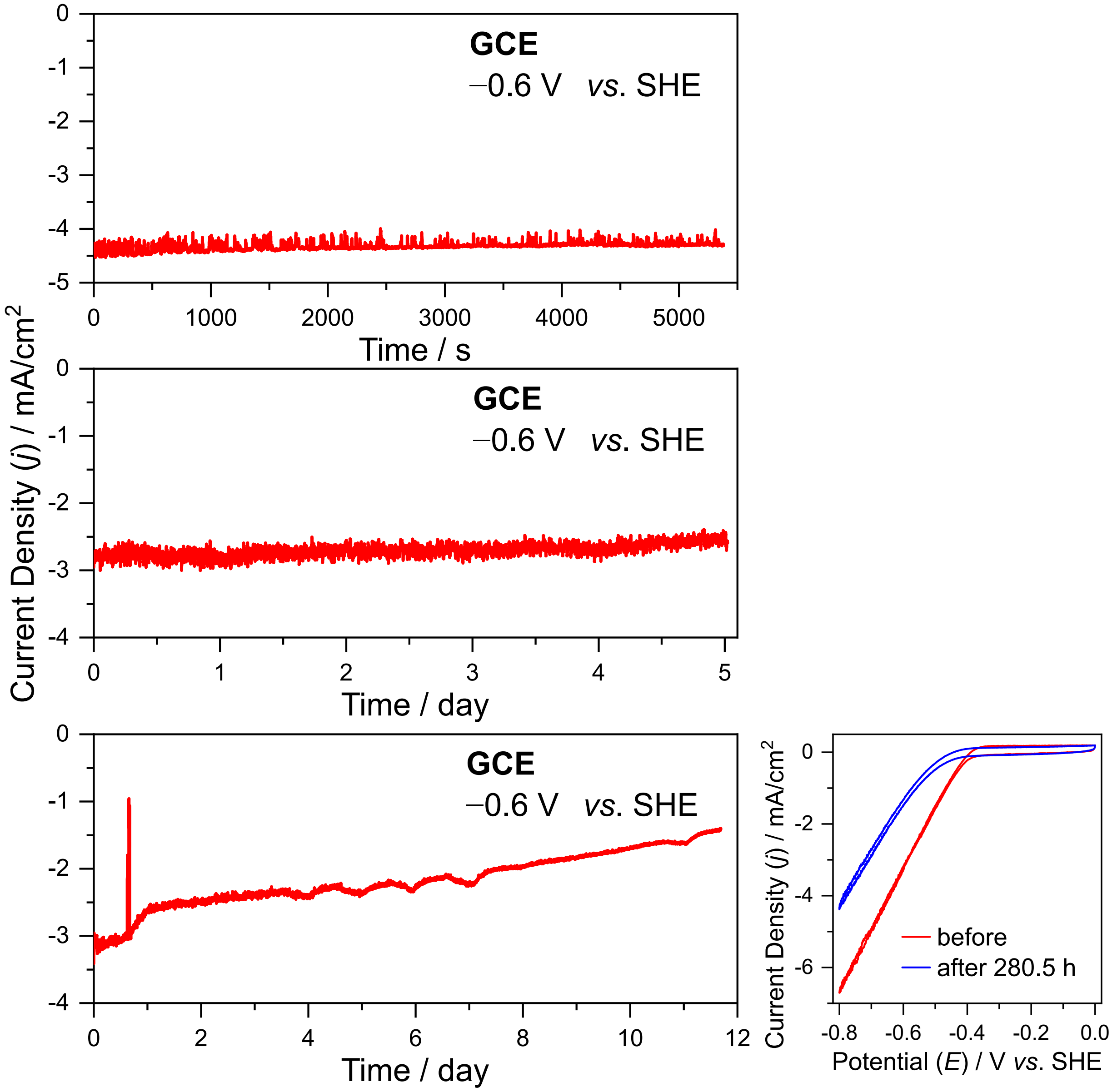

Figure S9: Additional stability tests for 1.5 h, 120.5 h, and 280.5 h. Corresponding 3rd CV scans before and after 280.5 h test.

S11 Bradford protein assay for hydrogenase leaching

To quantify the amount of hydrogenase leaching from the nanoITO electrode in the solution as a function of time, a micro-Bradford protein assay was performed. A nanoITO electrode was functionalized with hydrogenase (7.05 mg/ml) and dried under ambient conditions for 9 min. The functionalized electrode was immersed in buffer (stirred) and an aliquot of the solution was taken at 2, 10, and 30 min.

A bovine serum albumin (BSA) protein standard (200 mg/ml, Sigma-Aldrich) was used to create an adjusted calibration range between 1 and 5 μg/ml, while a Bradford dye (ITW Reagents, Switzerland) was used for protein staining. A 96 well plate was used for samples (1:1 ratio sample:dye) and shaken for 5 min in a microplate reader (ThermoScientific Multiskan FC), before the absorbance was recorded at 595 nm.

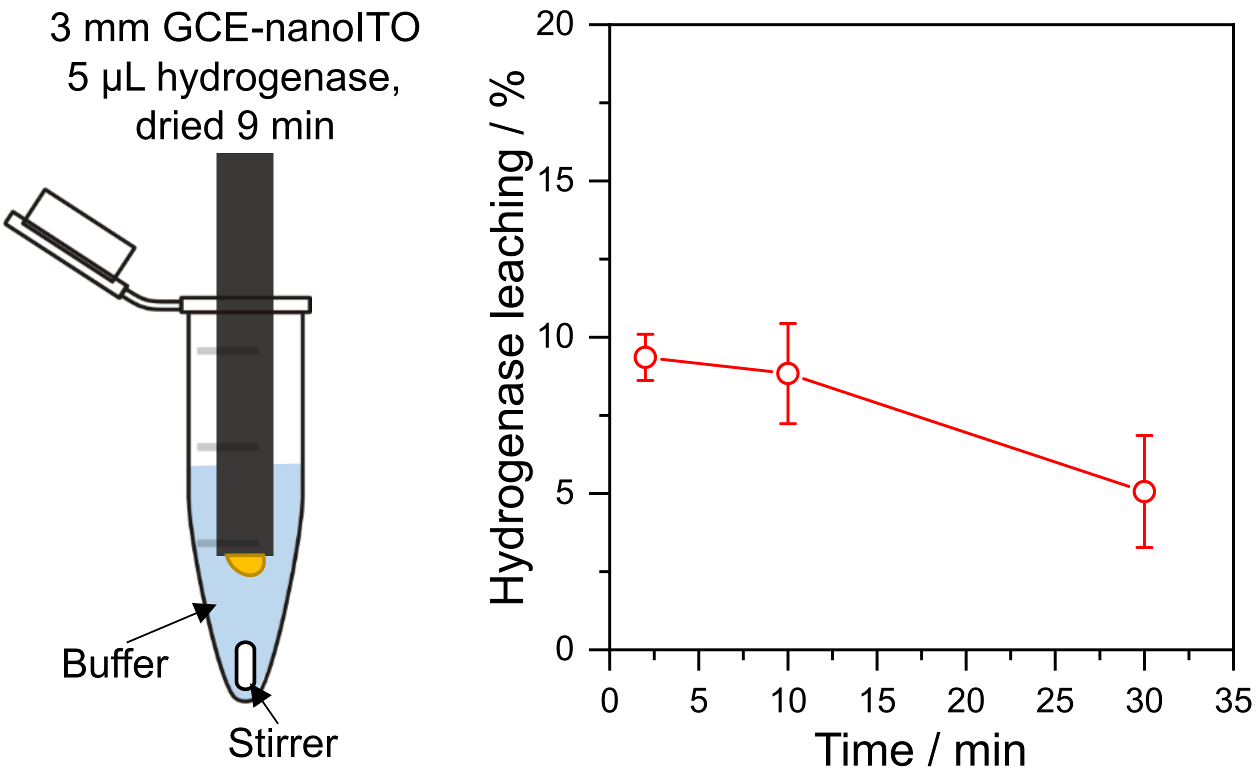

Figure S10: Bradford protein assay for hydrogenase leaching.

S12 Gas chromatography (GC) measurements and Faradaic efficiency (FE) determination

A temperature-controlled gas tight small volume cell was used (Adams & Chittenden Scientific Glass, BMS 956442) for electrochemical experiments in combination with gas chromatography (GC, SRI Instruments, 8610C). The cell was modified for 20 mm butyl rubber stoppers and detailed electrochemical setup can be found in the Supporting Information of our previous work.[S1] The GC employed a 5 Å molecular sieve column alongside a thermal conductivity detector and was equipped with 100 µL injection loop. A close-loop tubing circuit (PEEK) was installed from the small volume cell into the GC injection module (Figure S11). A peristaltic pump (Ismatec, REGLO Analog) continuously circulated the gas with a speed of approximately 2.7 mL/min. The gas in the cell was sampled and a chromatograph recorded each 5 min. Over pressure in the gas tight circuit was prevented with a PEEK tubing going from the cell into a vial containing water.

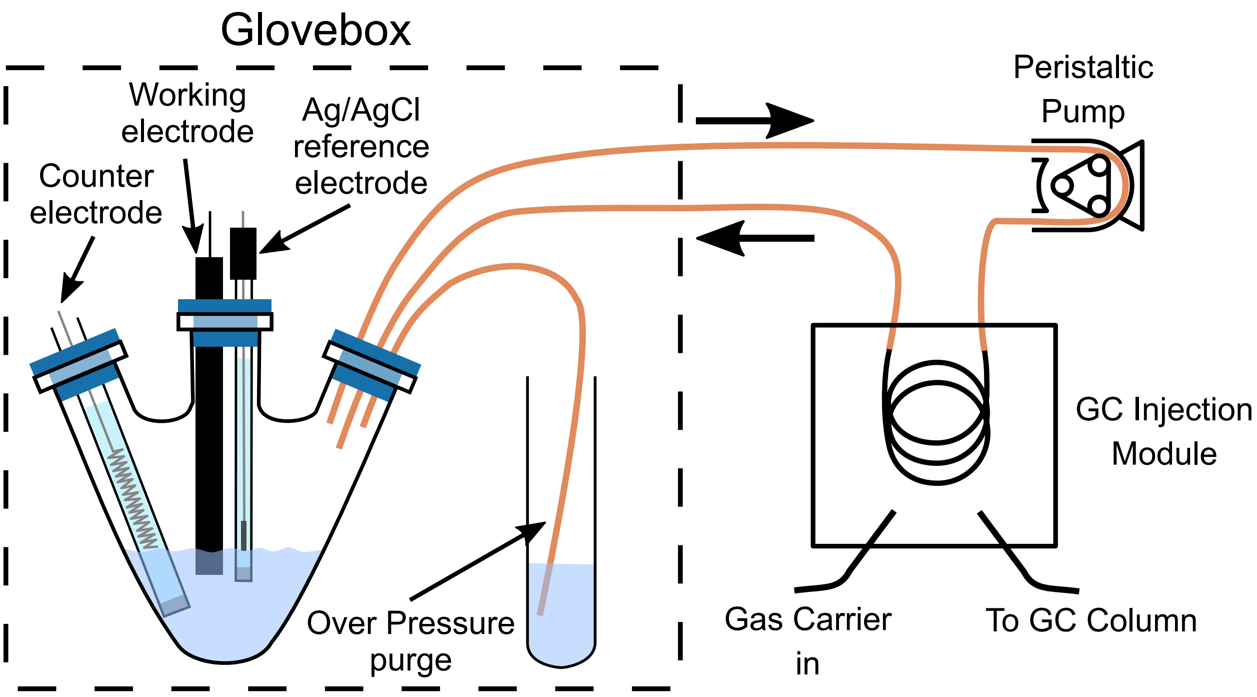

Figure S11: Sketch of the experimental setup used for the Faradaic efficiency measurements.

The linear response of the setup was verified by injecting given amounts of molecular hydrogen in the gas tight cell and subsequently measuring the hydrogen GC peak area. Figure S12 clearly shows the linear relation between the hydrogen injected amount and the measured peak area. The solid line represents the best fit to the experimental points.

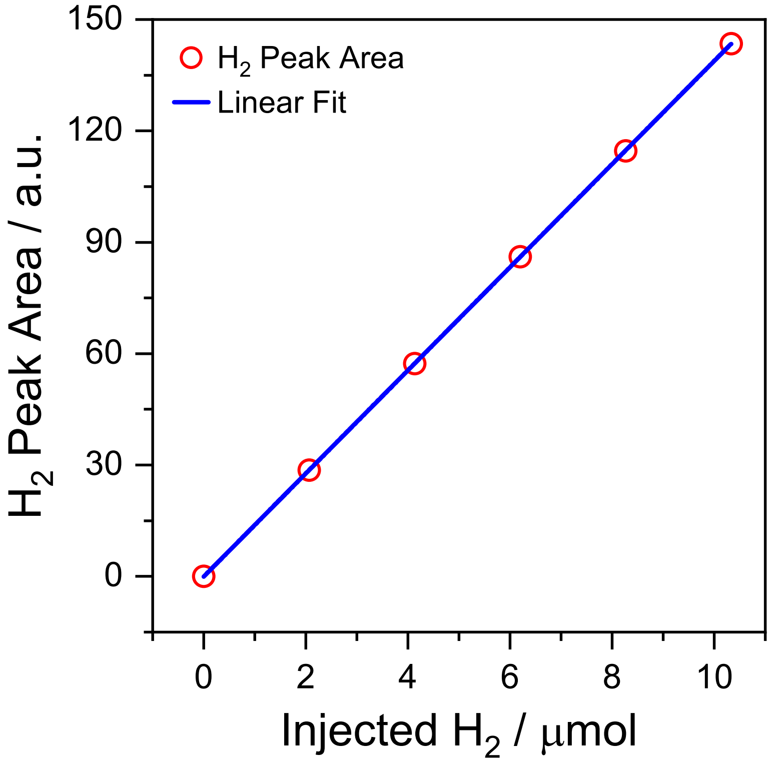

Figure S12: Linear response of the GC setup. The blue solid line represents the best fit to the experimental points (red circles).

For the Faradaic efficiency (FE) determination, the cell temperature was stabilized at 30 °C and the electrolysis was initiated simultaneously to the GC acquisition. A potential of −0.6 V *vs.* SHE was applied for 1.5 h. The current passing through the electrode was continuously monitored and GC spectra were recorded each 5 min. At the end of each experiment, a 100 µL injection of molecular hydrogen was performed to calibrate the response of the GC. The FE was then calculated by comparing the number of hydrogen moles produced (GC peak area) with the expected amount of hydrogen as determined by the electric current time-integration and applying the Faraday's laws of electrolysis. The measurement was repeated 3 times and the FE was found to be 98.5 ± 3.6 %.

S13 Long-term Faradaic efficiency of the CpI-nanoITO bioelectrode

Figure 2b in the main article confirms that the current density for H_2 production remains stable over 5 days. During this revision stage of publishing this work, the Faradaic efficiency of the CpI-nanoITO bioelectrode was also confirmed over a 5-day period of continuous potentiostatic operation. The previously employed gas-tight cell[S1] used in Figure 2b broke and a secondary gas-tight cell was employed. These experiments revealed the importance of efficient agitation of the electrochemical cell during continuous H_2 production. Figure S13a reports the evolution of the current density recorded over 5 days of continuous potentiostatic operation, using the cell which was no-longer gas-tight (broken), precluding the determination of Faradaic efficiency. Figure S13 reports the evolution of the current density (also recorded over 5 days) and Faradaic efficiency using a gas-tight cell in which good agitation conditions could not be achieved. It is important to note that the current density continuously decreased over 5 days. We hypothesize that this could be due to poor mass transport in the vicinity of the nanoITO-CpI electrode, which could result in a relatively basic pH (in comparison with the bulk) and the potential deactivation of CpI. Nevertheless, the Faradaic efficiency for H_2 production was determined to be 102.9 ± 1.4% (n = 7 GC readings of the same samples headspace) after 5 days of continuous operation. Figure S13c (photo) shows the accumulation of gas bubbles (presumably H_2) in this cell, as well as the sub-parr cell design that ultimately impedes efficient agitation. We conclude that efficient agitation (for H^+ delivery and H_2 removal) is critical to the operation of this nanoITO-CpI bioelectrodes.

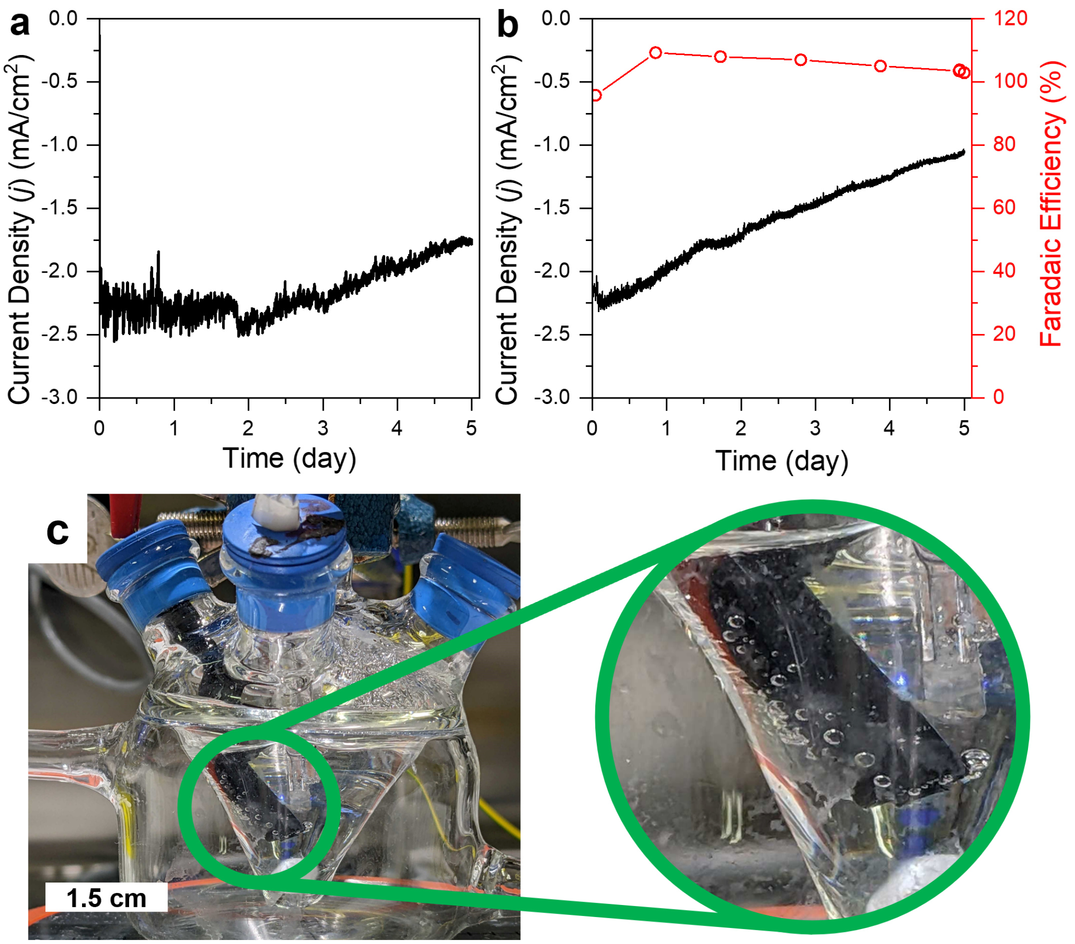

Figure S13: (a) Amperometric j–t curve of a GCE-nanoITO-hydrogenase bioelectrode at –0.6 V *vs.* SHE over 120 hours of continuous operation. Despite efficient stirring, this cell was not gas-tight. (b) Amperometric j–t curve of a GCE-nanoITO-hydrogenase bioelectrode at –0.6 V *vs.* SHE over 120 hours of continuous operation. This cell was technically gas-tight, although efficient stirring could not be achieved. The right-hand y-axis reports the Faradaic efficiency of this experiment (for H2) production, as determined by manual GC-TCD injections at the indicated times. Single injections were performed with the exception of the final data point (7 injection repeats). (c) Photograph of the cell used in (b), where H_2 bubble accumulation can be seen. The narrow conical design of this cell impedes efficient agitation using a magnetic stirrer.

S14 Inverted rotating disk electrode (iRDE) setup

The performance of the nanoITO hydrogenase was evaluated using an inverted RDE (iRDE) setup recently reported by the Broekmann group.[S7,S8] High-purity Ar 5.0 gas (99.9999%, Carbagas) was used to purge the electrochemical cell prior to use. A nano-ITO functionalized 5 mm glassy carbon RDE tip was introduced to the iRDE setup and the cell was first flushed with Ar. 5 µL of hydrogenase ($\sim$ 25 µg/0.4 nmol per 3 mm GCE) was drop cast onto the nanoITO functionalized electrode and dried under constant Ar flow. Approximately 80 mL of buffered electrolyte (100 mM MOPS buffer, pH 7) was introduced to the cell using a double-ended needle and overpressure of Ar. The main compartment of the cell contained a Ag/AgCl (3 M KCl) reference electrode, and a 3 cm^2 indium counter electrode was placed in a second compartment (separated by a FuelCellStore Nafion 212 membrane) containing the same buffered electrolyte. All potentials were converted to SHE by the following conversion factor: $E_{\mathrm{SHE}} = E_{\mathrm{Ag/AgCl}} + 0.21$ V.[S4] The main compartment of the cell was continuously flushed with Ar 5.0 using a mass-flow controller (21.4 mL/min), and the outflow of the cell was fed to a GC-thermal conductivity detector (GC-TCD, SRI instruments) for the quantification of H_2. The iRDE was rotated at 500 rpm. Experiments were performed at room temperature.

Figure S14 shows the photograph of inverted RDE (iRDE) and electrochemical cell assembly.

a working electrode
b RDE tip
c H-type cell body
d membrane and sealing junction
e purging gas inlet
f purging gas outlet (GC collection)
g reference electrode
h counterelectrode (Ir foil)

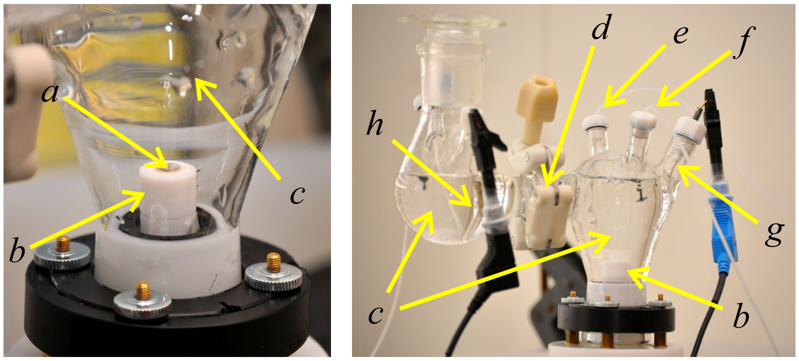

Figure S14: Inverted RDE (iRDE) and electrochemical cell assembly.

A video demonstrating the working condition of the iRDE setup has been included in the Supporting Information.

Figure S15 shows the schematic of iRDE setup.

a upper radial shaft seal

b working electrode

c RDE head/tip

d upper ceramic fitting

e ball bearing

f contact node

g pressuring gas inlet

h pressurized chamber

i lower radial shaft seal

j lower ceramic fitting

k metal shaft

l POM housing

m PTFE holder grooves

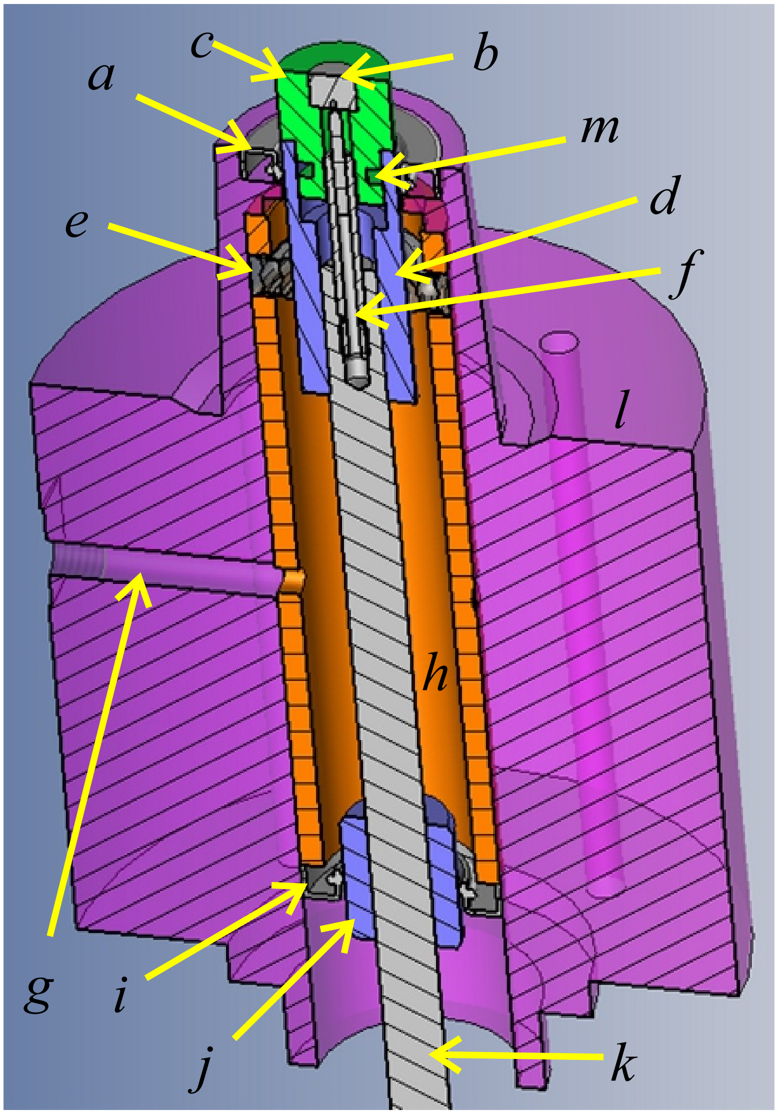

Figure S15: Cross-sectional representation of the iRDE setup. The H-type cell fitting on top of the instrument is connected to the counter electrode, reference electrode, feeding gas inlet and a gas chromatograph for gas product analysis through tight stationary sealed connections. POM and PTFE stand for polyoxymethylene and polytetrafluoroethylene, respectively.

S15 SDS-PAGE

The protein was analyzed for its purity and molecular weight by SDS-PAGE analysis. mPAGE™ 10% Bis-Tris Precast Gels were used. The protein was denatured using 0.1 M dithiothreitol (DTT) and by heating the mixture in 4× loading dye at 70 °C for 10 min before loading onto the gel. The gel was run in 1× SDS-PAGE Running Buffer at 180 V as the final voltage. The gel was stained with 1× Biotium One-Step Blue® Protein Gel Stain, and then imaged on Vilber Fusion FX imager.

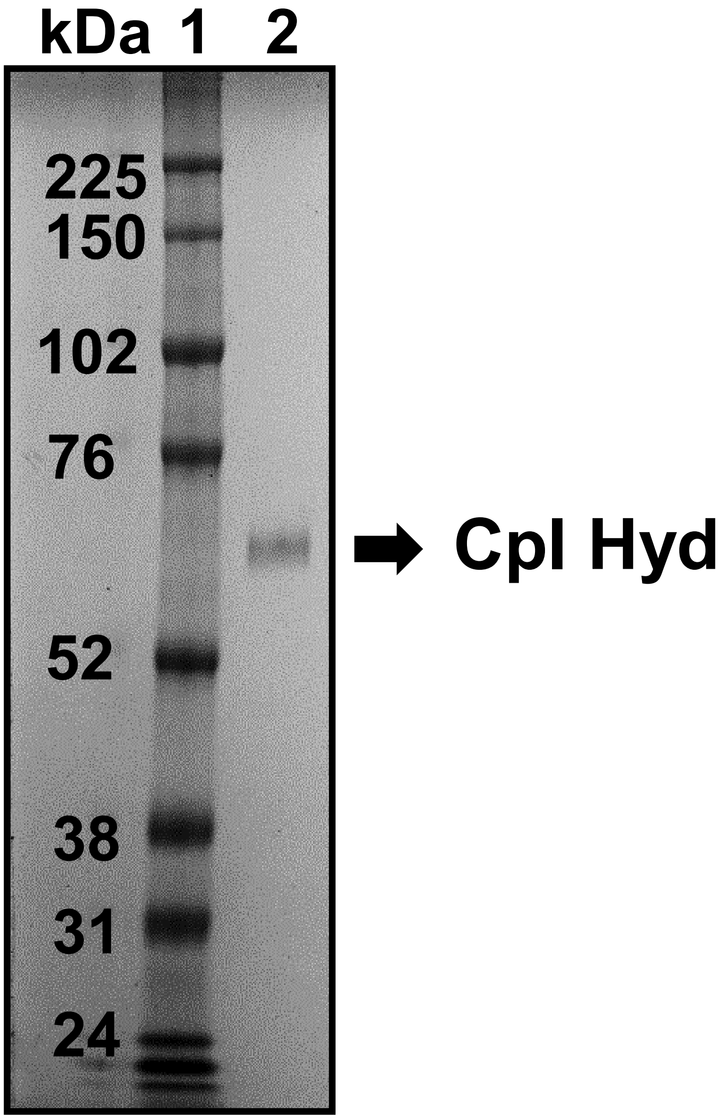

Figure S16: SDS-PAGE (10% acrylamide) of strep-tagged CpI. Lane 1 = Protein marker (Rainbow™ Marker RPN800E, kDa), lane 2 = purified CpI, 1 µg of protein.

References

(S1) Khushvakov, J.; Nussbaum, R.; Cadoux, C.; Duan, J.; Stripp, S. T.; Milton, R. D. Following Electroenzymatic Hydrogen Production by Rotating Ring–Disk Electrochemistry and Mass Spectrometry. *Angew. Chem. Int. Ed.* **2021**, *60*, 10001–10006.

(S2) Kuchenreuther, J. M.; Grady-Smith, C. S.; Bingham, A. S.; George, S. J.; Cramer, S. P.; Swartz, J. R. High-yield expression of heterologous [FeFe] hydrogenases in *Escherichia coli*. *PloS one* **2010**, *5*, e15491.

(S3) Hoertz, P. G.; Chen, Z.; Kent, C. A.; Meyer, T. J. Application of high surface area tin-doped indium oxide nanoparticle films as transparent conducting electrodes. *Inorg. Chem.* **2010**, *49*, 8179–8181.

(S4) Bard, A. J.; Faulkner, L. R. *Electrochemical Methods: Fundamentals and Applications, 2nd Edition*; John Wiley & Sons, 2000.

(S5) Reisner, E.; Powell, D. J.; Cavazza, C.; Fontecilla-Camps, J. C.; Armstrong, F. A. Visible light-driven H_2 production by hydrogenases attached to dye-sensitized TiO_2 nanoparticles. *J. Am. Chem. Soc.* **2009**, *131*, 18457–18466.

(S6) Reisner, E.; Fontecilla-Camps, J. C.; Armstrong, F. A. Catalytic electrochemistry of a [NiFeSe]-hydrogenase on TiO_2 and demonstration of its suitability for visible-light driven H_2 production. *Chem. Commun.* **2009**, 550–552.

(S7) Moreno-García, P.; Kovacs, N.; Grozovski, V.; Galvez-Vazquez, M. d. J.; Vesztergom, S.; Broekmann, P. Toward CO_2 electroreduction under controlled mass flow conditions: a combined inverted RDE and gas chromatography approach. *Anal. Chem.* **2020**, *92*, 4301–4308.

(S8) Moreno-García, P.; Grozovski, V.; Vázquez, M. d. J. G.; Mysuru, N.; Kiran, K.; Kovács, N.; Hou, Y.; Vesztergom, S.; Broekmann, P. Inverted RDE (iRDE) as novel test bed for studies

on additive-assisted metal deposition under gas-evolution conditions. *J. Electrochem. Soc.* **2020**, *167*, 042503.
